# Association of Sodium Thiosulfate With Risk of Ototoxic Effects From Platinum-Based Chemotherapy: A Systematic Review and Meta-analysis

**DOI:** 10.1001/jamanetworkopen.2021.18895

**Published:** 2021-08-02

**Authors:** Chih-Hao Chen, Chii-Yuan Huang, Heng-Yu Haley Lin, Mao-Che Wang, Chun-Yu Chang, Yen-Fu Cheng

**Affiliations:** 1 Department of Otolaryngology**-**Head and Neck Surgery, Taipei Veterans General Hospital, Taipei, Taiwan; 2 Faculty of Medicine, National Yang Ming Chiao Tung University, Taipei, Taiwan; 3 Department of Medical Education, Taipei Veterans General Hospital, Taipei, Taiwan; 4 Department of Anesthesiology, Taipei Tzu Chi Hospital, Buddhist Tzu Chi Medical Foundation, New Taipei City, Taiwan; 5 Department of Medical Research, Taipei Veterans General Hospital, Taipei, Taiwan; 6 Institute of Brain Science, National Yang Ming Chiao Tung University, Taipei, Taiwan

## Abstract

**Importance:**

Platinum-induced ototoxic effects are a significant issue because platinum-based chemotherapy is one of the most commonly used therapeutic medications. Sodium thiosulfate (STS) is considered a potential otoprotectant for the prevention of platinum-induced ototoxic effects that functions by binding the platinum-based agent, but its administration raises concerns regarding the substantial attenuation of the antineoplastic outcome associated with platinum.

**Objective:**

To evaluate the association between concurrent STS and reduced risk of ototoxic effects among patients undergoing platinum-based chemotherapy and to evaluate outcomes, including event-free survival, overall survival, and adverse outcomes.

**Data Sources:**

From inception through November 7, 2020, databases, including the Cochrane Library, PubMed, Embase, Web of Science, and Scopus, were searched.

**Study Selection:**

Studies enrolling patients with cancer who were undergoing platinum-based chemotherapy that compared ototoxic effects development between patients who received STS and patients who did not and provided adequate information for meta-analysis were regarded as eligible. This study followed the Preferred Reporting Items for Systematic Reviews and Meta-analyses (PRISMA) guidelines.

**Data Extraction and Synthesis:**

The data were extracted by 2 reviewers independently. A random-effects model was used to explore objectives.

**Main Outcomes and Measures:**

Relative risks (RRs) for ototoxic effects development and hemopoietic event development comparing the experimental group and the control group were estimated. Secondary outcomes were hazard ratios (HRs) for event-free survival and overall survival. Sensitivity analysis and trial sequential analysis were conducted to further consolidate pooled results.

**Results:**

Among 4 eligible studies that were included, there were 3 randomized clinical trials and 1 controlled study. A total of 278 patients were allocated to the experimental group (ie, platinum-based chemotherapy plus STS; 158 patients, including 13 patients using contralatral ears of the control group as samples) or the control group (ie, chemotherapy; 133 patients, including 13 patients using contralateral ears of the experimental group as samples). Overall, patients who received STS had a statistically significantly decreased risk of ototoxic effects during the course of platinum-based chemotherapy (RR, 0.61; 95% CI, 0.49-0.77; *P* < .001; *I*^2^ = 5.0%) without a statistically significant increase in the risk of poor event-free survival (HR, 1.13; 95% CI, 0.70-1.82; *P* = .61; *I*^2^ = 0%) or overall survival (HR, 1.90; 95% CI, 0.90-4.03; *P* = .09; *I*^2^ = 0%). In the trial sequential analysis of event-free survival (*z* = −0.52) and overall survival (*z* = −1.68), although the cumulative *z* curves did not surpass the traditional significance boundary (−1.96 to 1.96 for both) or sequential monitoring boundary (event-free survival: −8.0 to 8.0; overall survival boundary not renderable in the analysis because the information size was too small) of the adjusted CI, they did not reach the required information size.

**Conclusions and Relevance:**

This meta-analysis found that concurrent STS delivery was associated with a decreased risk of platinum-induced ototoxic effects among patients treated with platinum-induced chemotherapy. These findings suggest that concurrent STS for protection against ototoxic effects should be considered for patients indicated for platinum-based chemotherapy.

## Key Points

**Question:**

Is the use of sodium thiosulfate associated with decreased risk of ototoxic effects among patients treated with platinum-induced chemotherapy?

**Findings:**

In this meta-analysis of 4 clinical trials, including 3 randomized clinical trials and 278 patients, sodium thiosulfate (STS) was associated with a decreased risk of ototoxic effects when administered during the course of platinum-based chemotherapy.

**Meaning:**

This finding suggests that the prophylactic use of STS should be considered when platinum-based chemotherapy is indicated and that further large-scale trials are essential for solid application.

## Teaser

This systematic review and meta-analysis investigates 4 studies for the association of sodium thiosulfate with reduced risk of ototoxic effects among patients treated with platinum-induced chemotherapy.

## Introduction

Platinum-based chemotherapy is one of the most commonly used therapeutic agents in the treatment of solid tumors in pediatric and adult patients, including those with non–small cell lung carcinoma, ovarian cancer, hepatoblastoma, and head and neck cancer.^1,2,3,4^ However, the adverse outcomes associated with platinum, including nephrotoxic effects, myelosuppression, and ototoxic effects, limit its use and may be associated with long-term comorbidities in these patients.^5^ While medical intervention for prevention of nephrotoxic effects has been well illustrated, the prevention of platinum-induced ototoxic effects remains stagnant.^6,7,8^ Studies^9,10^ from 2005 and 2017 found that platinum can be sustained in the cochlea indefinitely, which is associated with long-term otologic complications, such as sensorineural hearing loss, tinnitus, and vestibulopathy. On the basis of the well-illustrated mechanism of platinum-induced ototoxic effects, studies identifying possible otoprotectants have been conducted in recent decades.^8,11,12,13^

Among otoprotective agents, the most studied^14,15,16^ has been amifostine, an aminothiol prodrug that is dephosphorylated to an active thiol that acts as a reactive oxygen scavenger. Although the association of amifostine with protection against nephrotoxic effects has been demonstrated, amifostine has failed to show an otoprotective association in previous studies.^16,17,18,19^ On the other hand, sodium thiosulfate (STS) has emerged as a promising otoprotectant. By protecting cells from apoptosis, STS has been found to be otoprotective in previous studies.^20,21,22^ However, concern that STS attenuates the antineoplastic outcome remains because 1 of the mechanisms of this medication is covalently binding to cisplatin;^23,24^ therefore, the transtympanic approach of STS has been attempted to avoid systemic influence on the antineoplastic outcome of platinum-based chemotherapy, although the results remain inconclusive.^25,26^ Additionally, under the circumstance that STS may potentially be associated with the antitumor efficacy of cisplatin, survival analysis is also needed to apply the medication safely in the clinic.

Regarding these issues, this study aimed to systematically review the current literature on the association of STS with the prevention of platinum-induced toxic effects. To explore the association of STS with the development of ototoxic effects and oncologic-related survival and the association of concurrent factors with these outcomes, we identified controlled studies and performed a meta-analysis to evaluate the efficacy of STS among patients with cancer undergoing platinum-based chemotherapy.

## Methods

This systematic review and meta-analysis followed the Preferred Reporting Items for Systematic Reviews and Meta-analyses (PRISMA) reporting guideline.^27^ The patient data used in this systematic review and meta-analysis were deidentified.

### Study Inclusion

Included studies were selected according to the following criteria: the study compared STS with a control regarding the outcome of interest (ie, development of ototoxic effects), the study clearly defined ototoxic effects, and the study provided an account of adequate information to quantify the effect estimates for meta-analysis. Studies were required to meet all conditions.

### Search Strategy and Identification of Eligible Studies

From inception through November 7, 2020, we searched databases, including the Cochrane Library, PubMed, Embase, Web of Science, and Scopus. We used a consolidation of Medical Subject Headings (MeSH) and text words to create 3 subsets of citations, 1 including studies of platinum-based treatment (ie, cisplatin, carboplatin, oxaliplatin, nedaplatin, triplatin tetranitrate, and satraplatin), the second including hearing complications (ie, hearing loss, hearing impairment, and ototoxicity), and the third including sodium thiosulfate treatment (ie, sodium thiosulfate). The detailed search strategy is displayed in eTable 1 in the Supplement. The identified records were screened by titles, abstracts, and keywords. Records with potential eligibility were then obtained for full-text review.

After review of the full texts by 2 authors (C.H.C. and C.Y.C.), the effect estimates of interest were extracted by consensus. Criteria were that the primary data should rate ototoxic effects development in STS and control groups, while the definition of ototoxic effects should be clearly reported in the eligible studies. The secondary outcomes of interest were event-free survival and overall survival. Event-free survival was defined as being free of relapse or progression of disease, diagnosis of a second primary tumor, or death. Additionally, adverse outcomes reported and assessed in studies were extracted.

### Data Management

The data from patients were allocated into 1 of 2 treatment groups: patients receiving STS while undergoing platinum-based chemotherapy (ie, the STS group) and patients undergoing platinum-based chemotherapy without STS administration (ie, the control group). The relative risk (RR) of ototoxic effects development and adverse outcomes consisting of hemopoietic events were computed comparing the groups. Hazard ratio (HR) was applied in survival analysis in the groups. The revised Cochrane risk of bias tool 2 was used to assess the methodological quality of the included studies.

### Statistical Analysis

The random-effects model, assuming that a second source of error other than sampling error existed, was used for effect size calculation. Concurrently, for data reported as graphical outcomes, we used WebPlotDigitizer data-extraction software version 4.3 (Ankit Rohatgi) to digitize graphs and extract data. The reliability of WebPlotDigitizer has been previously validated.^28^ The Cochran *Q* statistic and the *I*^2^ statistic were used to assess statistical heterogeneity. The heterogeneity was considered low, moderate, or high for *I*^2^ values of less than 50%, 50% to 74%, and 75% or greater, respectively.^29^ Additionally, sensitivity analyses were performed to test the strength of the results by excluding the non–randomized clinical trial (RCT), excluding carboplatin-based chemotherapy, excluding the study using the Brock grading system, excluding the study enrolling a small sample size, and examining every incidence of hematopoietic events after each chemotherapy cycle in the study by Freyer et al.^21^ All calculations for the meta-analysis were performed using R statistical software version 4.0.3 (R Project for Statistical Computing) in RStudio statistical software version 1.3.959 (RStudio) with the metaphor package. In addition, trial sequential analysis (TSA) was performed to evaluate whether the result would be subject to type I or type II errors associated with sparse data and a lack of power. This was done under the assumption that STS contributes to an RR decrease of 20% for the development of ototoxic effects and adverse outcomes and using Trial Sequential Analysis software version 0.9.5.10 beta (Copenhagen Trial Unit, Centre for Clinical Intervention).^30,31^ The models for all outcomes were based on an α value of .05 and a power of 80% using a 2-sided statistical test. The traditional significance boundary in TSA analysis was −1.96 to 1.96, and the sequential monitory boundary varied by analysis. Data were analyzed from November through December 2020.

## Results

Among 542 records in the preliminary search after removing 276 duplicates and screening by title and abstract, 10 studies underwent full-text review. We excluded 6 studies owing to a lack of comparison with the control group, insufficient data for meta-analysis, or lack of availability of the full text. As a result, 4 eligible studies, including 3 RCTs^21,22,25^ and 1 controlled study,^32^ were included (eFigure 1 in the Supplement). A total of 278 patients were allocated to the experimental group (ie, platinum-based chemotherapy plus STS; 158 patients, including 13 patients using contralateral ears of the control group as samples) or the control group (ie, chemotherapy; 133 patients, including 13 patients using contralateral ears of the experimental group as samples). The Brock grading system was used to define ototoxic effects in 1 study^22^ (hearing level ≥40 dB for both ears at grade I: 8 kHz; grade II: 8 kHz and 4 kHz; grade III: 8 kHz, 4 kHz, and 2 kHz; and grade IV: 8 kHz, 4 kHz, 2 kHz, and 1 kHz^33^), while 3 studies used American Speech-Language-Hearing Association criteria to define ototoxic effects^21,25,32^ (ie, >20 dB decrease at 1 test frequency or >10 dB decrease at 2 continuous test frequencies compared with a reference range baseline at the pure-tone threshold^34^). Patients undergoing cisplatin-based chemotherapy were enrolled in 3 studies,^21,22,25^ while 1 study^32^ included patients undergoing carboplatin-based chemotherapy. Patients younger than age 18 years were enrolled in 2 studies,^21,22^ while 2 studies^25,32^ enrolled patients older than age 18 years. Administration of STS was intravenous in 3 studies,^21,22,32^ and 1 study^25^ performed transtympanic injection to apply STS to the middle ear. In 2 studies,^21,22^ the adverse outcomes consisting of hematopoietic events were assessed and reported (Table). Further information regarding the included study population, regimen, and control groups is presented in eTable 2 in the Supplement.

**Table.** Study Characteristics

Study	Study type	Treatment	Patients, No. (No. STS/No. control	Event rate, No. events/No. total	Sex, No. men/No. women	Age, mean, y	Control group	Ototoxic effects scale	Adverse events
Brock et al,^22^ 2018	RCT	Cisplatin	101 (55/46)	STS group: 18/55	59/50	<18	Chemotherapy only	Brock grading^33^	Neutrophilia; thrombocytopenia; anemia
				Control group: 29/46					
Freyer et al,^21^ 2017	RCT	Cisplatin	104 (49/55)	STS group: 19/49	76/49	<18	Chemotherapy only	ASHA criteria^34^	Neutrophilia; thrombocytopenia; anemia
				Control group: 31/55					
Rolland et al,^25^ 2019	RCT	Cisplatin	13 (13/13)^a^	STS group: 8/13	10/3	>18	Chemotherapy only	ASHA criteria	NR
				Control group: 9/13					
Doolittle et al,^32^ 2001	Controlled clinical trial	Carboplatin	60 (41/19)	STS group: 18/41	29/31	>18	Chemotherapy only	ASHA criteria	NR
				Control group: 16/19					

Abbreviations: ASHA, American Speech-Language-Hearing Association; NR, not reported; RCT, randomized clinical trial; STS, sodium thiosulfate.

^a^ Studies used ears as the effect size of the study.

### Risk of Bias Assessment

Risk of bias was assessed in each included study (eFigure 2 and 3 in the Supplement). The randomization process was not mentioned in 1 study,^32^ while the allocation of patients was not concealed in another study.^25^ The length of follow-up was not reported in 2 studies,^25,32^ while 4 studies^21,22,25,32^ did not report whether the measurement or ascertainment of the outcome differed between intervention groups. Regarding all of the above, some concern remained regarding the risk of bias.

### Overall Association of STS With Prevention of Ototoxic Effects

**Figure 1.**
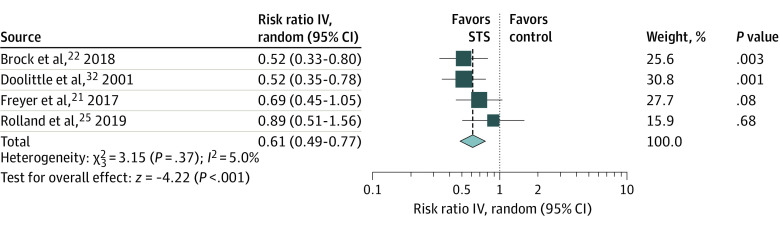
Overall Association of Sodium Thiosulfate (STS) With Prevention of Ototoxic Effects IV indicates inverse variance method.

The 4 included studies compared the risk of ototoxic effects development between patients who received STS with platinum-based chemotherapy and patients who did not receive STS with platinum-based chemotherapy.^21,22,25,32^ In overall pooled results, patients who underwent STS treatment with chemotherapy had a statistically significantly decreased risk of developing ototoxic effects (RR, 0.61; 95% CI, 0.49 to 0.77; *P* < .001; *I*^2^ = 5.0%) (Figure 1). The TSA of the overall association (z = 4.22) indicated a conclusive result given that the pooled sample size of 291 samples had reached the estimated required information size (RIS; 91 samples) and surpassed the traditional significance boundary and the sequential monitoring boundary (−1.96 to 1.96) of the adjusted CI favoring STS based on the a priori assumption that STS contributed a relative risk decrease of 20% (eFigure 4 in the Supplement).

### Association of STS With Event-Free Survival and Overall Survival

**Figure 2.**
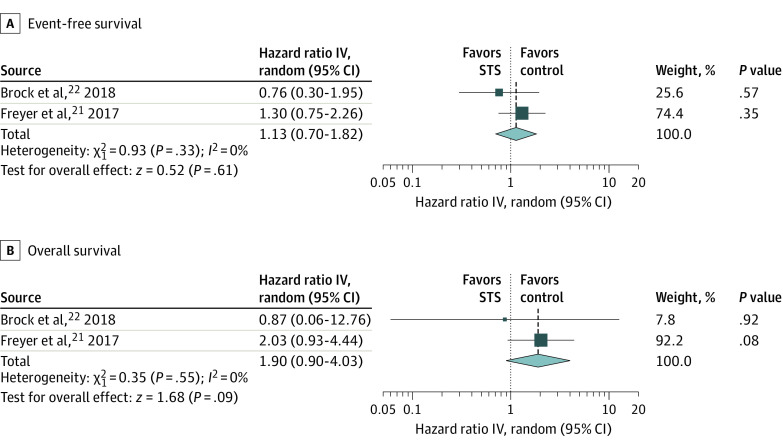
Event-Free Survival and Overall Survival IV indicates inverse variance method; STS, sodium thiosulfate.

In 2 studies that performed survival analyses, including event-free survival and overall survival,^21,22^ pooled association estimates of event-free survival showed no statistically significant difference (HR, 1.13; 95% CI, 0.70 to 1.82; *P* = .61; *I*^2^ = 0%) (Figure 2A). For overall survival, pooled association estimates also revealed no statistically significant difference (HR, 1.90; 95% CI, 0.90 to 4.03; *P* = .09; *I*^2^ = 0%) (Figure 2B). In the TSA of event-free survival among a pooled sample of 233 patients, although the cumulative *z* curve (*z* = −0.52) did not pass the traditional significance boundary or the sequential monitoring boundary (−8.00 to 8.00) of the adjusted CI, it had not yet reached the RIS of 3241 samples. Similarly, the cumulative *z* curve for overall survival (*z* = −1.68) surpassed neither the traditional significance boundary nor the sequential monitoring boundary (not renderable in the analysis because the information size was too small), but the pooled sample size of 233 samples did not reach the RIS of 8062 samples (eFigures 5 and 6 in the Supplement).

### Subgroup Analysis of the Association of STS With Ototoxic Effects by Administration Route

**Figure 3.**
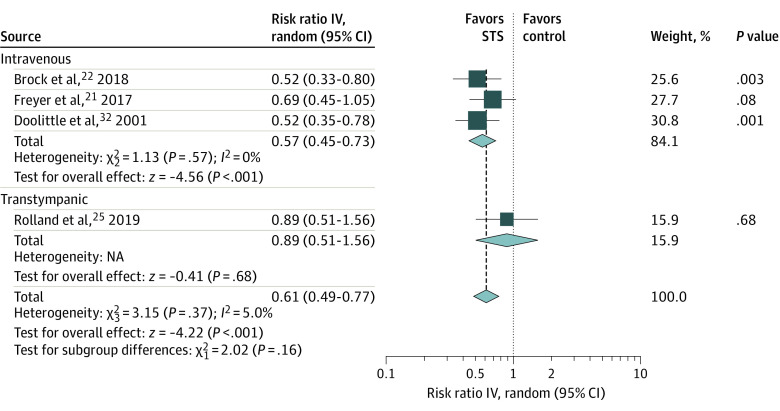
Subgroup Analysis by Delivery Route IV indicates inverse variance method; NA, not applicable; STS, sodium thiosulfate.

In the pooled estimates of 3 studies that administered STS via the intravenous route,^21,22,32^ STS was associated with decreased risk of ototoxic effects compared with not receiving STS (RR, 0.57; 95% CI, 0.45 to 0.73; *P* < .001; *I*^2^ = 0%) (Figure 3). In 1 study,^25^ STS was injected into the middle ear via the transtympanic route, and there was a statistically nonsignificant difference in ototoxic effects (RR, 0.89; 95% CI, 0.51 to 1.56; *P* = .68) (Figure 3). The TSA of the association of intravenous STS with ototoxic effects (*z* = 4.56) indicated a conclusive result, with the pooled result size of 265 patients reaching the estimated RIS of 87 samples and surpassing the traditional significance boundary and sequential monitoring boundary (−1.96 to 1.96) of the adjusted CI favoring STS based on the a priori assumption that STS contributes a relative risk decrease of 20% (eFigure 7 in the Supplement).

### Subgroup Analysis of the Association of STS With Ototoxic Effects by Age Group

**Figure 4.**
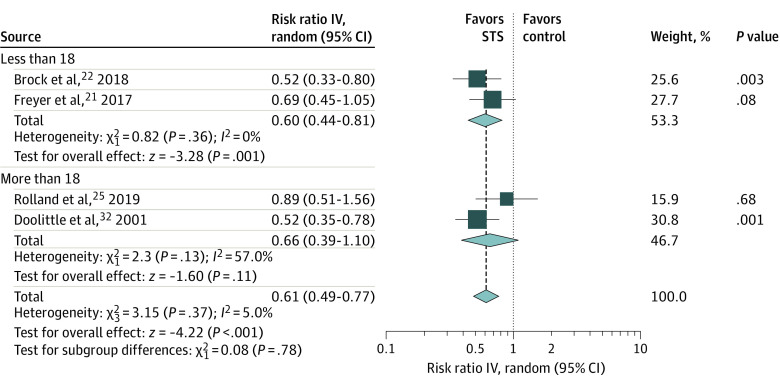
Subgroup Analysis by Age Group IV indicates inverse variance method; STS, sodium thiosulfate.

Younger patients undergoing cisplatin-based chemotherapy were enrolled in 2 studies,^21,22^ and in the pooled result, STS was associated with a decreased risk of ototoxic effects (RR, 0.60; 95% CI, 0.44 to 0.81; *P* = .001; *I*^2^ = 0%) (Figure 4). In pooled results of 2 studies enrolling older patients,^25,32^ there was no statistically significant difference in the risk of ototoxic effects development (RR, 0.66; 95% CI, 0.39 to 1.10; *P* = .11; *I*^2^ = 57.0%) (Figure 4). In the TSA of the STS association in the younger age group, the cumulative *z* curve (*z* = 3.28) surpassed the traditional significance boundary, but the sequential monitoring boundary could not be rendered because the first information fraction exceeded 100%, which indicated that the first study had obtained sufficient statistical power for a meta-analysis. Moreover, in the TSA of the association in the older age group, the cumulative *z* curve (*z* = 1.6) did not surpass the traditional significance boundary or sequential monitoring boundary (−3.42 to 3.42), but the pooled sample size of 86 samples did not reach the RIS of 221 samples or the inner wedge of futility (eFigures 8 and 9 in the Supplement).

### Association of STS With Hematopoietic Events

Hematopoietic adverse outcomes were reported in 2 studies.^21,22^ In the pooled estimate, there were statistically nonsignificant differences in the risk of development of neutropenia (RR, 1.00; 95% CI, 0.78 to 1.29; *P* = .97; *I*^2^ = 0%) (eFigure 10 in the Supplement), thrombocytopenia (RR, 0.94; 95% CI, 0.63 to 1.42; *P* = .78; *I*^2^ = 0%) (eFigure 11 in the Supplement), and anemia (RR, 0.88; 95% CI, 0.54 to 1.44; *P* = .61; *I*^2^ = 6.0%) (eFigure 12 in the Supplement). In the TSA of the association of STS with hematopoietic events, including neutropenia (z = −0.04), thrombocytopenia (z = −0.28), and anemia (z = 0.51), cumulative *z* curves did not surpass the traditional significance boundary or sequential monitoring boundaries (neutropenia and thrombocytopenia: −1.96 to 1.96; anemia: −2.63 to 2.63). In these 3 TSAs (pooled sample size of all results, 225 samples), the cumulative *z* score of neutropenia and thrombocytopenia reached the RIS (170 patients), while the cumulative *z* score of anemia (RIS = 365 samples) reached the inner wedge of futility (eFigures 13-15 in the Supplement).

### Sensitivity Analysis

After excluding the non-RCT, the study using carboplatin-based chemotherapy, the study using the Brock grading system, and the study with a small sample size, STS remained associated with a decreased risk of ototoxic effects development overall (eFigures 16-19 in the Supplement), while subsequent TSAs in these analyses further supported these results (eFigures 20-23 in the Supplement). Furthermore, in the influence analysis that involved leaving out each included study 1 at a time, the pooled estimates remained within the 95% CI of the overall pooled results for these outcomes (eFigure 24 in the Supplement). For hematopoietic events, after pooling the estimates of each cycle of chemotherapy in the study by Freyer et al,^21^ the results remained statistically nonsignificant for the development of neutropenia (eFigures 25-29 in the Supplement), thrombocytopenia (eFigures 30-34 in the Supplement), and anemia (eFigures 35-39 in the Supplement).

## Discussion

This meta-analysis found that STS use during the course of platinum-based chemotherapy was associated with decreased risk of ototoxic effects. Furthermore, in the pooled survival estimates, there was no statistically significant difference in event-free survival or overall survival between patients receiving STS and those not receiving STS. To our knowledge, this is the first meta-analysis that provides evidence for otoprotective medication against platinum-induced ototoxic effects.

### Comparison With Existing Literature

Several antioxidants are considered promising for preventing the toxic effects of platinum-based chemotherapy; however, none of them have been proven to be effective to date. Amifostine, a thiophosphate-reducing drug that acts as a reactive oxygen scavenger in normal tissues, has been studied the most.^8^ In a meta-analysis comprising 4 RCTs,^17^ the results failed to demonstrate the efficacy of amifostine for preventing cisplatin-induced hearing loss, although the results favored the experimental group with amifostine. Inadequate reporting of cases of ototoxic effects may contribute to underestimation of the outcome associated with the intervention. Furthermore, type II errors may also contribute to false negatives in the pooled estimate. Considering the above points, the present study included studies that used a clear definition of ototoxic effects to adequately extract the estimate and further confirmed the experimental outcomes by performing TSA.

### Pathophysiology of Platinum-Induced Ototoxic Effects

Several theoretical mechanisms account for platinum-induced ototoxic effects. Platinum medication has pharmacologic outcomes by inducing oxidative stress by generating reactive oxygen species (ROS) in tumor cells and by covalently binding with the DNA base of cells to impair antioxidative enzyme production.^5,35,36,37^ As a result, this medication prompts the apoptosis of tumor cells. These outcomes also play a key role in ototoxic effects. Studies have found that cisplatin easily accumulates in the cochlea indefinitely.^9,10^ Given that the cochlea is anatomically isolated and works as a closed system, the accumulation of cisplatin cannot be expelled in a timely manner,^9,10^ resulting in ROS overload and an impaired antioxidant system.^38^ The accumulation of ROS further causes mitochondrial membrane permeabilization and the apoptosis of cells in the stria vascularis, which would sustain degeneration, and this accumulated ROS further induces damage to the cochlear structure, including outer hair cells, inner hair cells, the stria vascularis, and even spiral ganglia, as the extent of cell damage broadens. Eventually, ototoxic effects develop owing to the long-term retention of platinum in the cochlea.^10,36,37,39,40,41^

### Evidence of Outcomes Associated With STS

Studies have illustrated the theoretical mechanism of STS. First, STS is a free-radical scavenger. When free radicals are produced by cisplatin toxic effects, they easily turn into ROS (eg, H_2_O_2_) at an extremely rapid rate, and STS directly reduces ROS.^6,42^ Second, STS binds to platinum and creates a biologically inactive complex.^43^ By eliminating free radicals produced by platinum toxic effects and inhibiting the outcome by binding platinum via covalent bonds, STS achieves an otoprotective outcome.

### Association of STS With Survival

Despite the otoprotection associated with STS, the attenuation outcome associated with forming covalent bonds raises another concern: systemic STS would substantially decrease the antineoplastic outcome associated with platinum.^7^ An in vitro study^44^ found that the delivery timing of STS matters in the antineoplastic efficacy of platinum and the otoprotective outcome of STS, given that STS may neutralize more platinum agent with greater persistence in the body. In pooled estimates of 2 studies^21,22^ that administered STS 6 hours after cisplatin infusion in our study, there was no statistically significant difference in event-free survival or overall survival.

### Subgroup Analysis of STS

To avoid the potential influence on the antitumor function of platinum, the transtympanic approach of STS has been investigated and has shown some efficacy in studies, mainly by the ability to form inactive complexes and prevent the release of cytochrome C in the inner ear.^26,45,46^ However, in the subgroup analysis of our study, we found that intravenous administration of STS was associated with an otoprotective outcome, while there was a decrease in ototoxic effects with administration via transtympanic injection, although this difference was not statistically significant. Various factors may attenuate the association of STS with otoprotective outcomes by transtympanic delivery. First, the permeability of the drug solution could be easily cofounded by other factors, including viscosity, environmental pH, or even the use of a facilitating agent.^46,47^ Second, because the administration would inevitably pass through the middle ear, lesions in the middle ear, such as effusions, would certainly influence the efficacy of STS. In the included study^25^ that enrolled patients undergoing the transtympanic approach of STS delivery, all patients had head and neck cancer and received concurrent chemoradiation therapy over the head and neck area. Under these circumstances, it is reasonable to speculate that the middle ear may sustain variable degrees of middle ear effusion owing to the nature of radiation to the eustachian tube area and eventually influence the outcome associated with STS via transtympanic delivery.

In the population with STS administration during cisplatin treatment course, younger age was associated with decreased risk of developing ototoxic effects. In pooled estimates in the older age group, there was still a decreased risk of ototoxic effects development, although the difference was not statistically significant. This may be partly associated with the lack of a statistically significant outcome with the transtympanic approach mentioned previously.

### Adverse Outcomes of STS

In the pooled estimate of 2 studies^21,22^ that reported hematopoietic adverse outcomes, including neutropenia, thrombocytopenia, and anemia, there was no statistically significant increase in the risk of myelosuppression. In contrast to STS being considered an agent associated with toxic effects of myelosuppression, some studies consider STS to be associated with protection against myelosuppression.^48,49^ However, the toxic effects of platinum on bone marrow may be associated not only with cytotoxic events on hematopoietic cells, but also with impairment of the sympathetic nervous system. The autonomic nerves in the bone marrow impaired by platinum also compromise hematopoietic stem cell mobilization and hematopoietic regeneration, and these outcomes are not preventable by STS.^50^ Under these circumstances, STS may not be associated with a protective outcome for myelosuppression. However, studies regarding other side effects, including electrolyte imbalance and gastrointestinal dysfunction (eg, nausea or vomiting), are absent. Therefore, further study is essential to explore the association of STS with hematopoietic events and obtain detailed adverse outcome data.

### Limitations

The study has several limitations. First, we did not exclude the non-RCT study. The pooled result may be subject to the unadjusted estimate of the non-RCT. We therefore conducted a sensitivity analysis by excluding the non-RCT study, and the pooled estimate in the sensitivity analysis remained statistically significant. Second, given that differences in baseline characteristics, including age, race, ethnicity, and underlying disease, across the studies may contribute to bias, we expect further studies to report efficacy according to different baseline characteristics. Third, the relatively small number of included studies may have affected the results of the subgroup analysis and the survival outcomes owing to insufficient statistical power. We suggest that further large-scale studies are essential to provide more detailed information and convincing results.

## Conclusions

We performed a meta-analysis to evaluate the association of STS with the prevention of platinum-induced ototoxic effects. We found that STS was associated with a decreased risk of ototoxic effects among patients treated with platinum-induced chemotherapy. However, investigating whether it is associated with an increased risk of poor event-free survival and overall survival requires further large-scale studies.

## Notes

Supplement. **eTable 1.** Detailed Search Strategy **eFigure 1.** Preferred Reporting Items for Systematic Reviews and Meta-analyses (PRISMA) Flow Diagram **eTable 2.** Detailed Study Characteristics **eFigure 2.** Risk of Bias **eFigure 3.** Percentage of Risk of Bias **eFigure 4.** Trial Sequential Analysis (TSA) of Overall Association of Sodium Thiosulfate (STS) **eFigure 5.** Trial Sequential Analysis (TSA) of Event-Free Survival **eFigure 6.** Trial Sequential Analysis (TSA) of Overall Survival **eFigure 7.** Trial Sequential Analysis (TSA) of the Association of Intravenous Sodium Thiosulfate (STS) **eFigure 8.** Trial Sequential Analysis (TSA) of the Sodium Thiosulfate (STS) Association in the Younger Age Group **eFigure 9.** Trial Sequential Analysis (TSA) of the Association in the Older Age Group **eFigure 10.** Association of Sodium Thiosulfate (STS) With Neutropenia **eFigure 11.** Association of Sodium Thiosulfate (STS) With Thrombocytopenia **eFigure 12.** Association of Sodium Thiosulfate (STS) With Anemia **eFigure 13.** Trial Sequential Analysis (TSA) of the Association of Sodium Thiosulfate (STS) With Neutropenia **eFigure 14.** Trial Sequential Analysis (TSA) of the Association of Sodium Thiosulfate (STS) With Anemia **eFigure 15.** Trial Sequential Analysis (TSA) of the Association of Sodium Thiosulfate (STS) With Thrombocytopenia **eFigure 16.** Sensitivity Analysis After Removal Of Non–Randomized Clinical Trial (RCT) **eFigure 17.** Sensitivity Analysis After Removal of Study Using Carboplatin-Based Chemotherapy **eFigure 18.** Sensitivity Analysis After Removal of Study Using Brock Grading System **eFigure 19.** Sensitivity Analysis After Removal of Study With Small Sample Size **eFigure 20.** Trial Sequential Analysis (TSA) of the Sensitivity Analysis After Removal of Non–Randomized Controlled Trial (RCT) **eFigure 21.** Trial Sequential Analysis (TSA) of Sensitivity Analysis After Removal Of Study Using Carboplatin-Based Chemotherapy **eFigure 22.** Trial Sequential Analysis (TSA) of Sensitivity Analysis After Removal of Study Using Brock Grading System **eFigure 23.** Trial Sequential Analysis (TSA) of Sensitivity Analysis After Removal of Study With Small Sample Size **eFigure 24.** Influence Analysis of Overall Association of Sodium Thiosulfate (STS) **eFigure 25.** Sensitivity Analysis of Sodium Thiosulfate (STS) Association With Neutropenia, Cycle 2 **eFigure 26.** Sensitivity Analysis of Sodium Thiosulfate (STS) Association With Neutropenia, Cycle 3 **eFigure 27.** Sensitivity Analysis of Sodium Thiosulfate (STS) Association With Neutropenia, Cycle 4 **eFigure 28.** Sensitivity Analysis of Sodium Thiosulfate (STS) Association With Neutropenia, Cycle 5 **eFigure 29.** Sensitivity Analysis of Sodium Thiosulfate (STS) Association With Neutropenia, Cycle 6 **eFigure 30.** Sensitivity Analysis of Sodium Thiosulfate (STS) Association With Thrombocytopenia, Cycle 2 **eFigure 31.** Sensitivity Analysis of Sodium Thiosulfate (STS) Association With Thrombocytopenia, Cycle 3 **eFigure 32.** Sensitivity Analysis of Sodium Thiosulfate (STS) Association With Thrombocytopenia, Cycle 4 **eFigure 33.** Sensitivity Analysis of Sodium Thiosulfate (STS) Association With Thrombocytopenia, Cycle 5 **eFigure 34.** Sensitivity Analysis of Sodium Thiosulfate (STS) Association With Thrombocytopenia, Cycle 6 **eFigure 35.** Sensitivity Analysis of Sodium Thiosulfate (STS) Association With Anemia, Cycle 2 **eFigure 36.** Sensitivity Analysis of Sodium Thiosulfate (STS) Association With Anemia, Cycle 3 **eFigure 37.** Sensitivity Analysis of Sodium Thiosulfate (STS) Association With Anemia, Cycle 4 **eFigure 38.** Sensitivity Analysis of Sodium Thiosulfate (STS) Association With Anemia, Cycle 5 **eFigure 39.** Sensitivity Analysis of Sodium Thiosulfate (STS) Association With Anemia, Cycle 6 eReferences
