# Supplementary material for: Association of Sodium Thiosulfate With Risk of Ototoxic Effects From Platinum-Based Chemotherapy: A Systematic Review and Meta-analysis
Source: JAMA Netw Open. 2021 Aug 2;4(8):e2118895. doi: 10.1001/jamanetworkopen.2021.18895 (PMC8329743; doi:10.1001/jamanetworkopen.2021.18895)
Supplement: Supplement. — eTable 1. Detailed Search Strategy eFigure 1. Preferred Reporting Items for Systematic Reviews and Meta-analyses (PRISMA) Flow Diagram eTable 2. Detailed Study Characteristics eFigure 2. Risk of Bias eFigure 3. Percentage of Risk of Bias eFigure 4. Trial Sequential Analysis (TSA) of Overall Association of Sodium Thiosulfate (STS) eFigure 5. Trial Sequential Analysis (TSA) of Event-Free Survival eFigure 6. Trial Sequential Analysis (TSA) of Overall Survival eFigure 7. Trial Sequential Analysis (TSA) of the Association of Intravenous Sodium Thiosulfate (STS) eFigure 8. Trial Sequential Analysis (TSA) of the Sodium Thiosulfate (STS) Association in the Younger Age Group eFigure 9. Trial Sequential Analysis (TSA) of the Association in the Older Age Group eFigure 10. Association of Sodium Thiosulfate (STS) With Neutropenia eFigure 11. Association of Sodium Thiosulfate (STS) With Thrombocytopenia eFigure 12. Association of Sodium Thiosulfate (STS) With Anemia eFigure 13. Trial Sequential Analysis (TSA) of the Association of Sodium Thiosulfate (STS) With Neutropenia eFigure 14. Trial Sequential Analysis (TSA) of the Association of Sodium Thiosulfate (STS) With Anemia eFigure 15. Trial Sequential Analysis (TSA) of the Association of Sodium Thiosulfate (STS) With Thrombocytopenia eFigure 16. Sensitivity Analysis After Removal Of Non–Randomized Clinical Trial (RCT) eFigure 17. Sensitivity Analysis After Removal of Study Using Carboplatin-Based Chemotherapy eFigure 18. Sensitivity Analysis After Removal of Study Using Brock Grading System eFigure 19. Sensitivity Analysis After Removal of Study With Small Sample Size eFigure 20. Trial Sequential Analysis (TSA) of the Sensitivity Analysis After Removal of Non–Randomized Controlled Trial (RCT) eFigure 21. Trial Sequential Analysis (TSA) of Sensitivity Analysis After Removal Of Study Using Carboplatin-Based Chemotherapy eFigure 22. Trial Sequential Analysis (TSA) of Sensitivity Analysis After Removal of Study Using Brock Grading Sy [file jamanetwopen-e2118895-s001.pdf]

Supplemental Online Content

Chen CH, Huang CY, Lin HYH, Wang MC, Chang CY, Cheng YF. Association of sodium thiosulfate with risk of ototoxic effects from platinum-based chemotherapy: a systematic review and meta-analysis. *JAMA Netw Open*. 2021;4(8):e2118895. doi:10.1001/jamanetworkopen.2021.18895

eTable 1. Detailed Search Strategy
eFigure 1. Preferred Reporting Items for Systematic Reviews and Meta-analyses (PRISMA) Flow Diagram
eTable 2. Detailed Study Characteristics
eFigure 2. Risk of Bias
eFigure 3. Percentage of Risk of Bias
eFigure 4. Trial Sequential Analysis (TSA) of Overall Association of Sodium Thiosulfate (STS)
eFigure 5. Trial Sequential Analysis (TSA) of Event-Free Survival
eFigure 6. Trial Sequential Analysis (TSA) of Overall Survival
eFigure 7. Trial Sequential Analysis (TSA) of the Association of Intravenous Sodium Thiosulfate (STS)
eFigure 8. Trial Sequential Analysis (TSA) of the Sodium Thiosulfate (STS) Association in the Younger Age Group
eFigure 9. Trial Sequential Analysis (TSA) of the Association in the Older Age Group
eFigure 10. Association of Sodium Thiosulfate (STS) With Neutropenia
eFigure 11. Association of Sodium Thiosulfate (STS) With Thrombocytopenia
eFigure 12. Association of Sodium Thiosulfate (STS) With Anemia
eFigure 13. Trial Sequential Analysis (TSA) of the Association of Sodium Thiosulfate (STS) With Neutropenia
eFigure 14. Trial Sequential Analysis (TSA) of the Association of Sodium Thiosulfate (STS) With Anemia
eFigure 15. Trial Sequential Analysis (TSA) of the Association of Sodium Thiosulfate (STS) With Thrombocytopenia
eFigure 16. Sensitivity Analysis After Removal of Non–Randomized Clinical Trial (RCT)
eFigure 17. Sensitivity Analysis After Removal of Study Using Carboplatin-

© 2021 Chen CH et al. *JAMA Network Open.*

Based Chemotherapy
eFigure 18. Sensitivity Analysis After Removal of Study Using Brock Grading System
eFigure 19. Sensitivity Analysis After Removal of Study With Small Sample Size
eFigure 20. Trial Sequential Analysis (TSA) of the Sensitivity Analysis After Removal of Non–Randomized Controlled Trial (RCT)
eFigure 21. Trial Sequential Analysis (TSA) of Sensitivity Analysis After Removal Of Study Using Carboplatin-Based Chemotherapy
eFigure 22. Trial Sequential Analysis (TSA) of Sensitivity Analysis After Removal of Study Using Brock Grading System
eFigure 23. Trial Sequential Analysis (TSA) of Sensitivity Analysis After Removal of Study With Small Sample Size
eFigure 24. Influence Analysis of Overall Association of Sodium Thiosulfate (STS)
eFigure 25. Sensitivity Analysis of Sodium Thiosulfate (STS) Association With Neutropenia, Cycle 2
eFigure 26. Sensitivity Analysis of Sodium Thiosulfate (STS) Association With Neutropenia, Cycle 3
eFigure 27. Sensitivity Analysis of Sodium Thiosulfate (STS) Association With Neutropenia, Cycle 4
eFigure 28. Sensitivity Analysis of Sodium Thiosulfate (STS) Association With Neutropenia, Cycle 5
eFigure 29. Sensitivity Analysis of Sodium Thiosulfate (STS) Association With Neutropenia, Cycle 6
eFigure 30. Sensitivity Analysis of Sodium Thiosulfate (STS) Association With Thrombocytopenia, Cycle 2
eFigure 31. Sensitivity Analysis of Sodium Thiosulfate (STS) Association With Thrombocytopenia, Cycle 3
eFigure 32. Sensitivity Analysis of Sodium Thiosulfate (STS) Association With Thrombocytopenia, Cycle 4
eFigure 33. Sensitivity Analysis of Sodium Thiosulfate (STS) Association With Thrombocytopenia, Cycle 5
eFigure 34. Sensitivity Analysis of Sodium Thiosulfate (STS) Association With Thrombocytopenia, Cycle 6
eFigure 35. Sensitivity Analysis of Sodium Thiosulfate (STS) Association With Anemia, Cycle 2
eFigure 36. Sensitivity Analysis of Sodium Thiosulfate (STS) Association With

© 2021 Chen CH et al. *JAMA Network Open.*

Anemia, Cycle 3
eFigure 37. Sensitivity Analysis of Sodium Thiosulfate (STS) Association With Anemia, Cycle 4
eFigure 38. Sensitivity Analysis of Sodium Thiosulfate (STS) Association With Anemia, Cycle 5
eFigure 39. Sensitivity Analysis of Sodium Thiosulfate (STS) Association With Anemia, Cycle 6
eReferences

This supplemental material has been provided by the authors to give readers additional information about their work.

© 2021 Chen CH et al. *JAMA Network Open.*

eTable 1. Detailed Search Strategy

Database	Query
PubMed	((((((((("Cisplatin"[Mesh]) OR ("Cisplatin/adverse effects"[Mesh] OR "Cisplatin/therapeutic use"[Mesh] OR "Cisplatin/toxicity"[Mesh])) OR "Carboplatin"[Mesh]) OR ("Carboplatin/adverse effects"[Mesh] OR "Carboplatin/therapeutic use"[Mesh] OR "Carboplatin/toxicity"[Mesh])) OR "Oxaliplatin"[Mesh]) OR ("Oxaliplatin/adverse effects"[Mesh] OR "Oxaliplatin/therapeutic use"[Mesh] OR "Oxaliplatin/toxicity"[Mesh])) OR "nedaplatin" [Supplementary Concept]) OR "BBR 3464" [Supplementary Concept]) OR "satraplatin" [Supplementary Concept] OR "Cisplatin"[TIAB] OR "cis-Diamminedichloroplatinum(II)"[TIAB] OR "Platinum "[TIAB] OR "Diamminodichloride"[TIAB] OR "cis-Platinum"[TIAB] OR "cis Platinum"[TIAB] OR "Dichlorodiammineplatinum"[TIAB] OR "cis-Diamminedichloroplatinum"[TIAB] OR "cis Diamminedichloroplatinum"[TIAB] OR "cis-Dichlorodiammineplatinum(II)"[TIAB] OR "Platino"[TIAB] OR "Platinol"[TIAB] OR "Biocisplatinum"[TIAB] OR "Platidiam"[TIAB] OR "Carboplatin"[TIAB] OR "cis-Diammine(cyclobutanedicarboxylato)platinum II"[TIAB] OR "Paraplatin"[TIAB] OR "Paraplatine"[TIAB] OR "Ribocarbo"[TIAB] OR "Carboplat"[TIAB] OR "Ercar"[TIAB] OR "Oxaliplatin"[TIAB] OR "1,2-Diamminocyclohexane(trans-1)oxolatoplatinum(II)"[TIAB] OR "Oxaliplatine"[TIAB] OR "Eloxatine"[TIAB] OR "Eloxatin"[TIAB] OR "Nedaplatin"[TIAB] OR "cis-diammine(glycolato)platinum II"[TIAB] OR "(glycolato-O,O')diammineplatinum II"[TIAB] OR "triplatin tetranitrate"[TIAB] OR "Satraplatin"[TIAB] OR "Cisplatin" OR "cis-Diamminedichloroplatinum(II)" OR "Platinum " OR "Diamminodichloride" OR "cis-Platinum" OR "cis Platinum" OR "Dichlorodiammineplatinum" OR "cis-Diamminedichloroplatinum" OR "cis Diamminedichloroplatinum" OR "cis-Dichlorodiammineplatinum(II)" OR "Platino" OR "Platinol" OR "Biocisplatinum" OR "Platidiam" OR "Carboplatin" OR "cis-Diammine(cyclobutanedicarboxylato)platinum II" OR "Paraplatin" OR "Paraplatine" OR "Ribocarbo" OR "Carboplat" OR "Ercar" OR "Oxaliplatin" OR "1,2-Diamminocyclohexane(trans-1)oxolatoplatinum(II)" OR "Oxaliplatine" OR "Eloxatine" OR "Eloxatin" OR "Nedaplatin" OR "cis-diammine(glycolato)platinum II" OR "(glycolato-O,O')diammineplatinum II" OR "triplatin tetranitrate" OR "Satraplatin") AND ((((("Ototoxicity/complications"[Mesh] OR "Ototoxicity/prevention and control"[Mesh])) OR "Ototoxicity"[Mesh]) OR "Hearing Loss"[Mesh]) OR ("Hearing Loss/complications"[Mesh] OR "Hearing Loss/diagnosis"[Mesh]) OR "Hearing loss"[TIAB] OR "Hearing Impairment"[TIAB] OR "ototoxicity"[TIAB] OR "Ototoxicities"[TIAB] OR "Drug-Induced Ototoxicity"[TIAB] OR "Drug Induced Ototoxicity"[TIAB] OR "Drug-Related Ototoxicity"[TIAB] OR "Drug Related Ototoxicity"[TIAB] OR "Hearing loss" OR "Hearing Impairment" OR "ototoxicity" OR "Ototoxicities" OR "Drug-Induced Ototoxicity" OR "Drug Induced Ototoxicity" OR "Drug-Related Ototoxicity" OR "Drug Related Ototoxicity") AND ("sodium thiosulfate" [Supplementary Concept] OR "Sodium thiosulfate"[TIAB] OR "sodium thiosulfate pentahydrate"[TIAB] OR "Sodium thiosulfate" OR "sodium thiosulfate pentahydrate")

© 2021 Chen CH et al. *JAMA Network Open.*

Embase	('cisplatin'/exp OR 'carboplatin'/exp OR 'oxaliplatin'/exp OR 'nedaplatin'/exp OR 'triplatin tetranitrate'/exp OR 'satraplatin'/exp) AND ('hearing impairment'/exp OR ototoxicities OR 'drug-induced ototoxicity' OR 'drug induced ototoxicity' OR 'ototoxicity'/exp OR 'ototoxic lesion' OR 'ototoxicity' OR 'ototoxicity prevention' OR 'toxicity, auditory' OR 'drug related ototoxicity' OR 'drug-related ototoxicity') AND ('sodium thiosulfate'/exp OR 'sodium thiosulfate pentahydrate')
Web of Science	TS=(("Cisplatin" OR "cis-Diamminedichloroplatinum(II)" OR "Platinum " OR "Diamminodichloride" OR "cis-Platinum" OR "cis Platinum" OR "Dichlorodiammineplatinum" OR "cis-Diamminedichloroplatinum" OR "cis Diamminedichloroplatinum" OR "cis-Dichlorodiammineplatinum(II)" OR "Platino" OR "Platinol" OR "Biocisplatinum" OR "Platidiam" OR "Carboplatin" OR "cis-Diammine(cyclobutanedicarboxylato)platinum II" OR "Paraplatin" OR "Paraplatine" OR "Ribocarbo" OR "Carboplat" OR "Ercar" OR "Oxaliplatin" OR "1,2-Diamminocyclohexane(trans-1)oxolatoplatinum(II)" OR "Oxaliplatine" OR "Eloxatine" OR "Eloxatin" OR "Nedaplatin" OR "cis-diammine(glycolato)platinum II" OR "(glycolato-O,O')diammineplatinum II" OR "triplatin tetranitrate" OR "Satraplatin")AND("Hearing loss" OR "Hearing Impairment" OR "ototoxicity" OR "Ototoxicities" OR "Drug-Induced Ototoxicity" OR "Drug Induced Ototoxicity" OR "Drug-Related Ototoxicity" OR "Drug Related Ototoxicity")AND("Sodium thiosulfate" OR "sodium thiosulfate pentahydrate"))
Scopus	(TITLE-ABS-KEY("Cisplatin" OR "cis-Diamminedichloroplatinum(II)" OR "Platinum " OR "Diamminodichloride" OR "cis-Platinum" OR "cis Platinum" OR "Dichlorodiammineplatinum" OR "cis-Diamminedichloroplatinum" OR "cis Diamminedichloroplatinum" OR "cis-Dichlorodiammineplatinum(II)" OR "Platino" OR "Platinol" OR "Biocisplatinum" OR "Platidiam" OR "Carboplatin" OR "cis-Diammine(cyclobutanedicarboxylato)platinum II" OR "Paraplatin" OR "Paraplatine" OR "Ribocarbo" OR "Carboplat" OR "Ercar" OR "Oxaliplatin" OR "1,2-Diamminocyclohexane(trans-1)oxolatoplatinum(II)" OR "Oxaliplatine" OR "Eloxatine" OR "Eloxatin" OR "Nedaplatin" OR "cis-diammine(glycolato)platinum II" OR "(glycolato-O,O')diammineplatinum II" OR "triplatin tetranitrate" OR "Satraplatin") AND TITLE-ABS-KEY("Hearing loss" OR "Hearing Impairment" OR "ototoxicity" OR "Ototoxicities" OR "Drug-Induced Ototoxicity" OR "Drug Induced Ototoxicity" OR "Drug-Related Ototoxicity" OR "Drug Related Ototoxicity") AND TITLE-ABS-KEY("Sodium thiosulfate" OR "sodium thiosulfate pentahydrate"))
Cochrane Library	ID Search<br>#1 MeSH descriptor: [Cisplatin] explode all trees<br>#2 MeSH descriptor: [Cisplatin] explode all trees and with qualifier(s): [adverse effects - AE]<br>#3 MeSH descriptor: [Cisplatin] explode all trees and with qualifier(s): [therapeutic use - TU]<br>#4 MeSH descriptor: [Cisplatin] explode all trees and with qualifier(s): [toxicity - TO]<br>#5 MeSH descriptor: [Carboplatin] explode all trees

© 2021 Chen CH et al. *JAMA Network Open.*

	#6 MeSH descriptor: [Carboplatin] explode all trees and with qualifier(s): [adverse effects - AE]<br>#7 MeSH descriptor: [Carboplatin] explode all trees and with qualifier(s): [therapeutic use - TU]<br>#8 MeSH descriptor: [Carboplatin] explode all trees and with qualifier(s): [toxicity - TO]<br>#9 MeSH descriptor: [Oxaliplatin] explode all trees<br>#10 MeSH descriptor: [Oxaliplatin] explode all trees and with qualifier(s): [adverse effects - AE]<br>#11 MeSH descriptor: [Oxaliplatin] explode all trees and with qualifier(s): [therapeutic use - TU]<br>#12 MeSH descriptor: [Oxaliplatin] explode all trees and with qualifier(s): [toxicity - TO]<br>#13 Cisplatin<br>#14 Diamminedichloroplatinum(II)<br>#15 Platinum<br>#16 Diamminodichloride<br>#17 cis-Platinum<br>#18 cis Platinum<br>#19 Dichlorodiammineplatinum<br>#20 cis-Diamminedichloroplatinum<br>#21 cis Diamminedichloroplatinum<br>#22 cis-Dichlorodiammineplatinum(II)<br>#23 Platino<br>#24 Platinol<br>#25 Biocisplatinum<br>#26 Platidiam<br>#27 Carboplatin<br>#28 cis-Diammine(cyclobutanedicarboxylato)platinum II<br>#29 Paraplatin<br>#30 Paraplatine<br>#31 Ribocarbo<br>#32 Carboplat<br>#33 Ercar<br>#34 Oxaliplatin<br>#35 Oxaliplatine<br>#36 Eloxatine<br>#37 Eloxatin<br>#38 Nedaplatin<br>#39 cis-diammine(glycolato)platinum II<br>#40 (glycolato-O,O')diammineplatinum II<br>#41 triplatin tetranitrate

© 2021 Chen CH et al. *JAMA Network Open.*

	#42 Satraplatin<br>#43 {OR #1-#42}<br>#44 MeSH descriptor: [Hearing Loss] explode all trees<br>#45 MeSH descriptor: [Hearing Loss] explode all trees and with qualifier(s): [complications - CO]<br>#46 MeSH descriptor: [Correction of Hearing Impairment] explode all trees<br>#47 MeSH descriptor: [Ototoxicity] explode all trees<br>#48 MeSH descriptor: [Ototoxicity] explode all trees and with qualifier(s): [complications - CO]<br>#49 MeSH descriptor: [Ototoxicity] explode all trees and with qualifier(s): [prevention & control - PC]<br>#50 Hearing loss<br>#51 Hearing Impairment<br>#52 ototoxicity<br>#53 Ototoxicities<br>#54 Drug-Induced Ototoxicity<br>#55 Drug Induced Ototoxicity<br>#56 Drug-Related Ototoxicity<br>#57 Drug Related Ototoxicity<br>#58 {OR #44-#57}<br>#59 MeSH descriptor: [Gold Sodium Thiosulfate] explode all trees<br>#60 Sodium thiosulfate<br>#61 sodium thiosulfate pentahydrate<br>#62 {OR #59-#61}<br>#63 #43 AND #58 AND #62

© 2021 Chen CH et al. *JAMA Network Open.*

eFigure 1. Preferred Reporting Items for Systematic Reviews and Meta-analyses (PRISMA) Flow Diagram

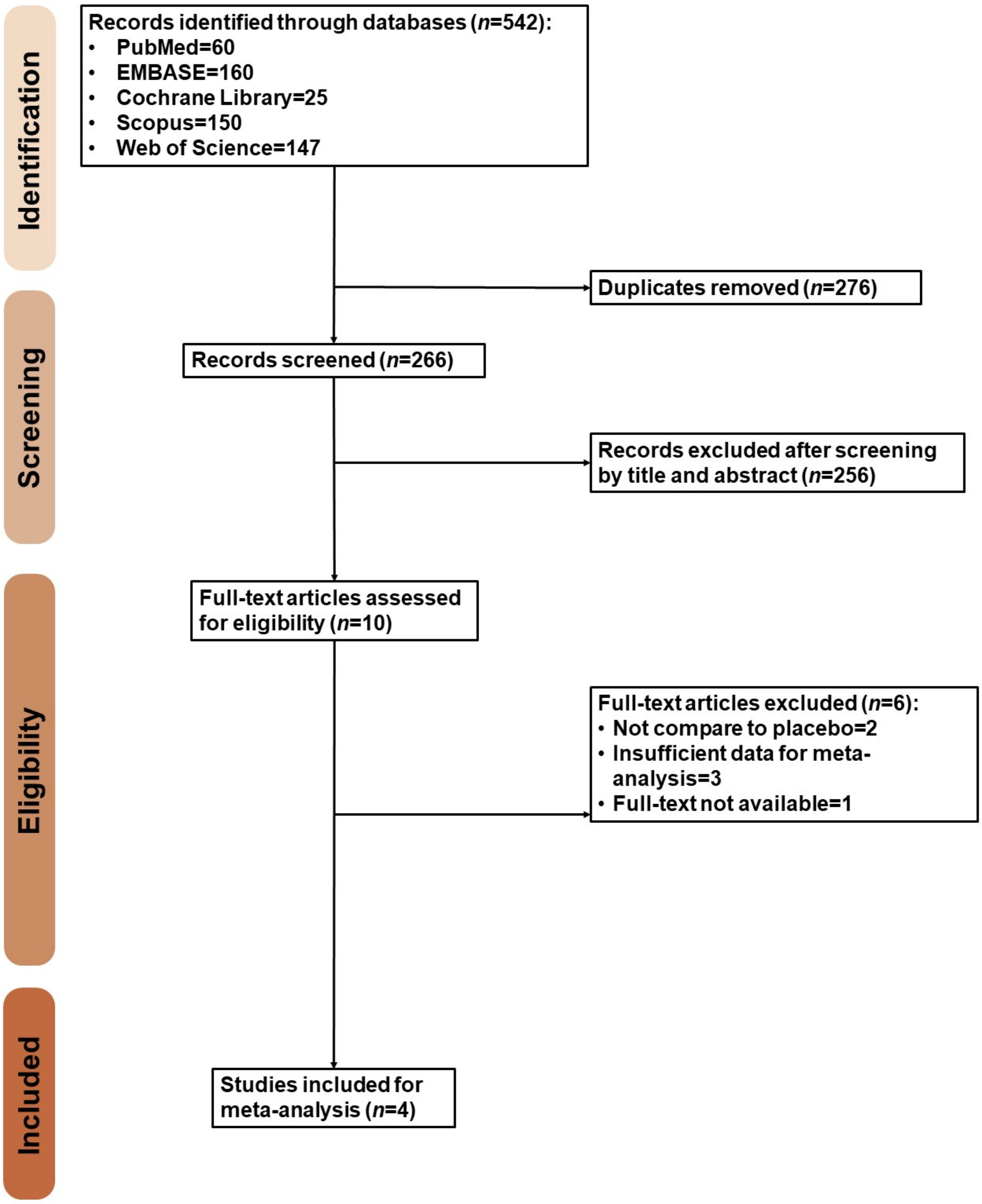

© 2021 Chen CH et al. *JAMA Network Open.*

eTable 2. Detailed Study Characteristics

Study	Disease	Intervention arm	Control arm	Dose
Brock et al,[1] 2018	Hepatoblastoma (localized)	Intravenously; 6 hours after the end of the cisplatin infusion.	Chemotherapy only	20 g/m^2
Doolittle et al,[2] 2001	CNS malignancy	Intravenously; Regimen *1*: 2h and 6h after the end of chemotherapy infusion. *Regimen 2.:* 4h and 8h after the end of chemotherapy infusion.	Chemotherapy only	20 g/m^2 then 16g/m^2
Freyer et al,[3] 2017	Multiple cancer	Intravenously; 6 hours after the end of each cisplatin infusion.	Chemotherapy only;	16 gm/m^2
Rolland et al,[4] 2019	Head and neck cancer	Trans-tympanic injection	Chemotherapy only	0.1ml of 0.5 M.STS gel

© 2021 Chen CH et al. *JAMA Network Open.*

eFigure 2. Risk of Bias

The revised Cochrane Risk of Bias Tool 2 was used to evaluate to quality of included studies.

Studies with intention-to-treat	Unique ID	Study ID	Experimental	Comparator	Outcome	Weight	Randomization process	Deviations from intended intervent	Missing outcome data	Measurement of the outcome	Selection of the reported result	Overall
	Brock et al., 2018	1	STS+cisplatin	cisplatin	Ototoxicity	1	+	+	+	?	+	!
	Doolittle et al., 2001	30	STS+carboplatin	carboplatin	Ototoxicity	1	?	?	+	?	+	!
	Freyer et al., 2017	100	STS+cisplatin	cisplatin	Ototoxicity	1	+	+	+	+	+	+
	Rolland et al, 2019	8	STS+cisplatin	cisplatin	Ototoxicity	1	?	+	+	?	+	!

\+ Low risk

? Some concerns

? High risk

© 2021 Chen CH et al. *JAMA Network Open.*

eFigure 3. Percentage of Risk of Bias

Overall, 70% of the studies are subject to some concerns of bias. Some concerns on measurement of the outcome remain in 70% of the studies. Some concerns on deviation from intended interventions remain in 25% of studies; 50% of the included studies sustain high risk on randomization process.

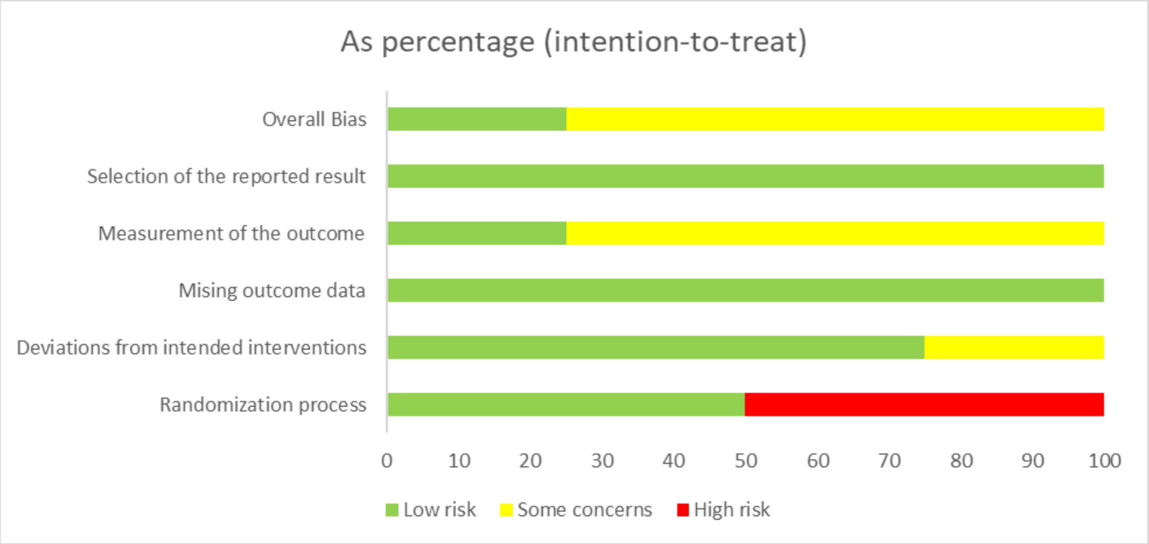

© 2021 Chen CH et al. *JAMA Network Open.*

eFigure 4. Trial Sequential Analysis (TSA) of Overall Association of Sodium Thiosulfate (STS)

A conclusive result with the cumulative Z-curve reaching the estimated required information size (RIS) and surpassing both the traditional significance boundary and the sequential monitoring boundary of the adjusted significance threshold that favored STS based on the a priori relative risk decrease of 20%.

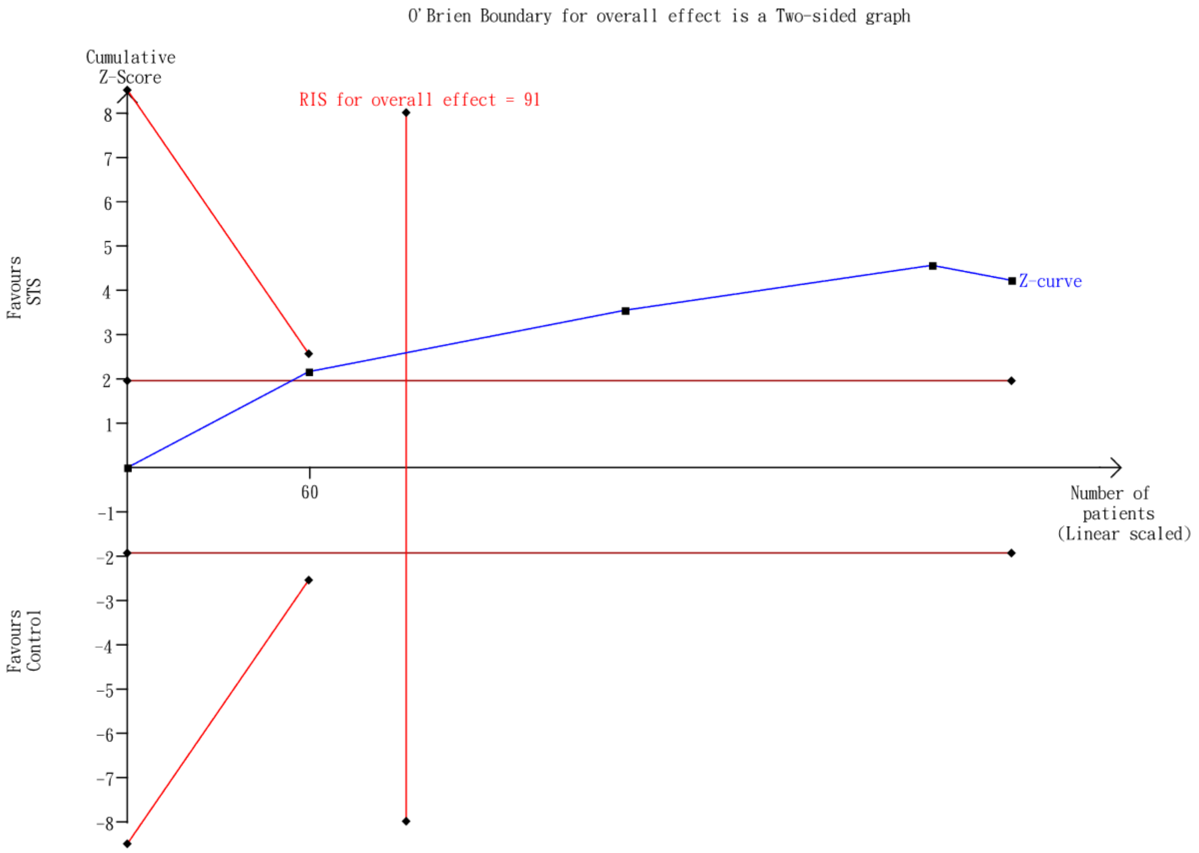

© 2021 Chen CH et al. *JAMA Network Open.*

eFigure 5. Trial Sequential Analysis (TSA) of Event-Free Survival
Although the cumulative Z-curve does not pass the traditional significance boundary and the sequential monitoring boundary of adjusted confidence interval, it hasn't reached the RIS yet. In other word, the non-significant result between STS group and control group remains inconclusive.

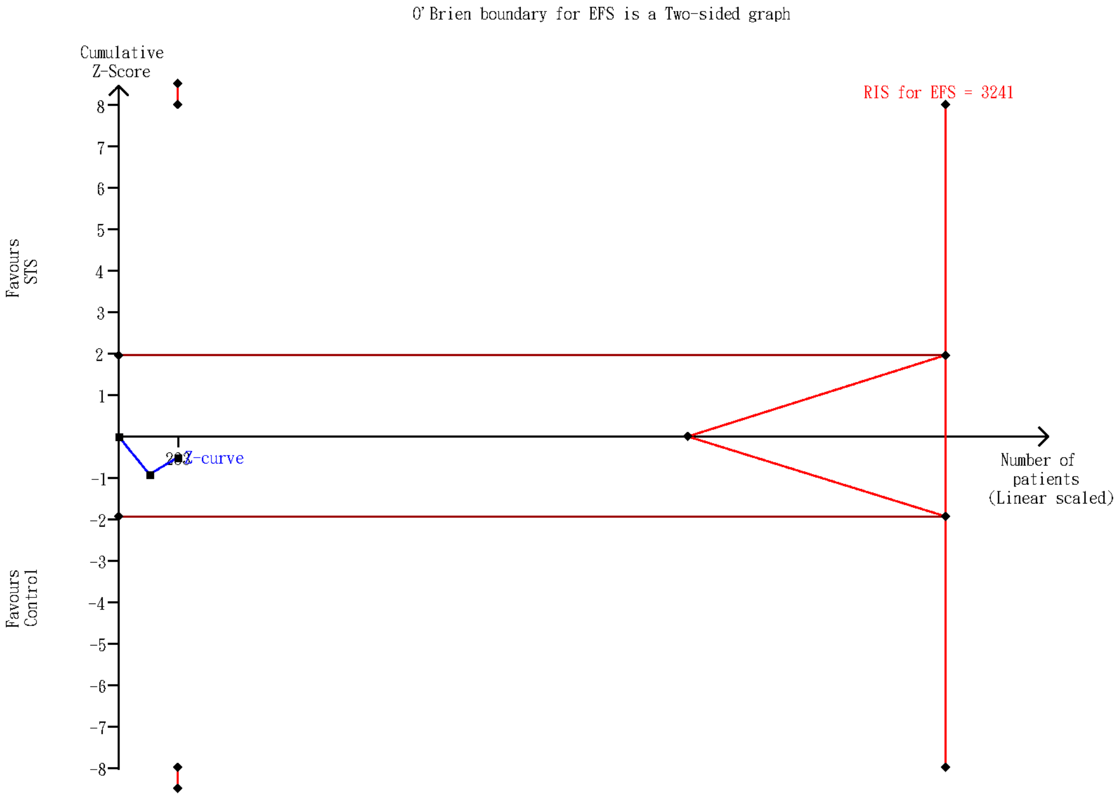

© 2021 Chen CH et al. *JAMA Network Open.*

eFigure 6. Trial Sequential Analysis (TSA) of Overall Survival
Although the cumulative Z-curve does not pass the traditional significance boundary and the sequential monitoring boundary of adjusted confidence interval, it hasn't reached the RIS yet. In other word, the non-significant result between STS group and control group remains inconclusive.

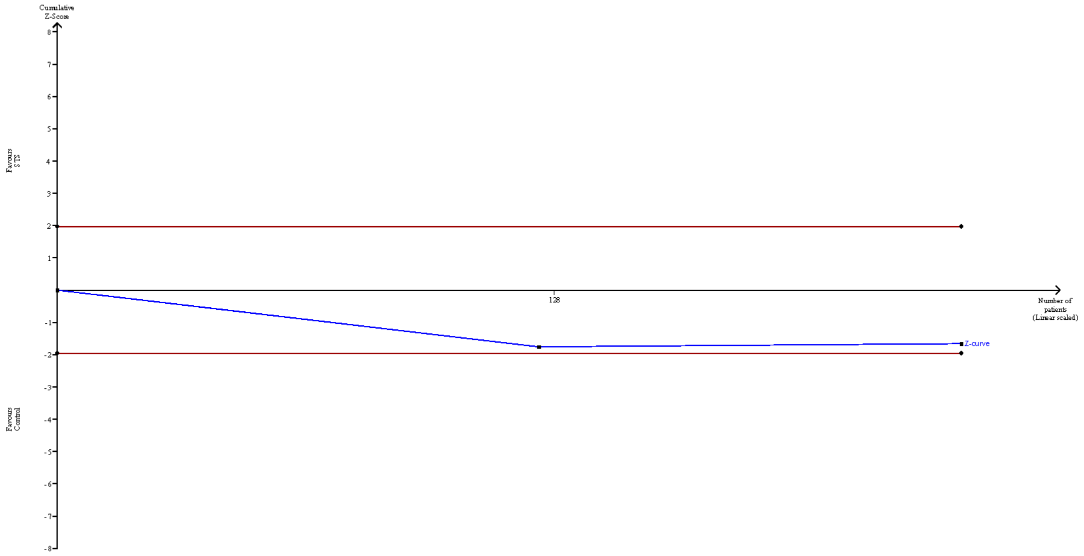

© 2021 Chen CH et al. *JAMA Network Open.*

eFigure 7. Trial Sequential Analysis (TSA) of the Association of Intravenous Sodium Thiosulfate (STS)

The cumulative Z-curve reached the estimated RIS and surpassed both the traditional significance boundary in and the sequential monitoring boundary of the adjusted significance threshold that favored STS based on the a priori relative risk decrease of 20%

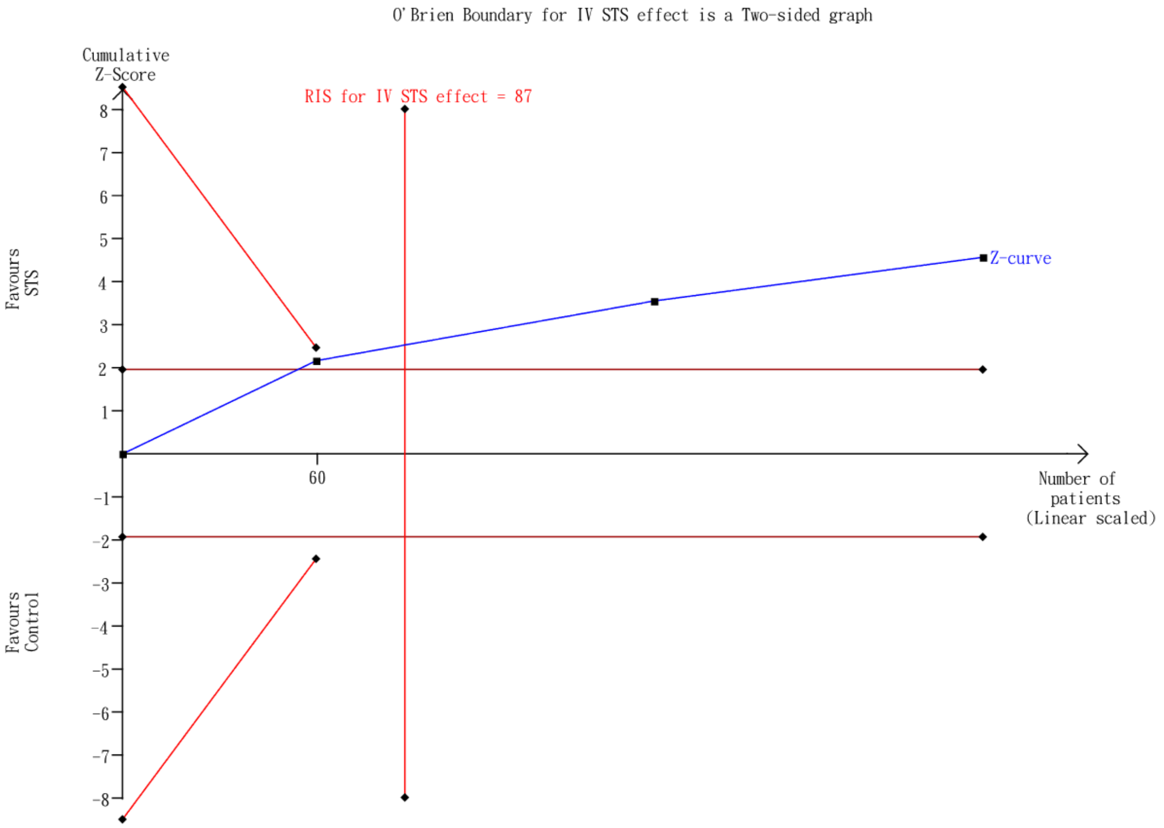

© 2021 Chen CH et al. *JAMA Network Open.*

eFigure 8. Trial Sequential Analysis (TSA) of the Sodium Thiosulfate (STS) Association in the Younger Age Group

The cumulative Z-curve surpassed the traditional significance boundary. The sequential monitoring boundary since the sample size of first study has reached estimated RIS.

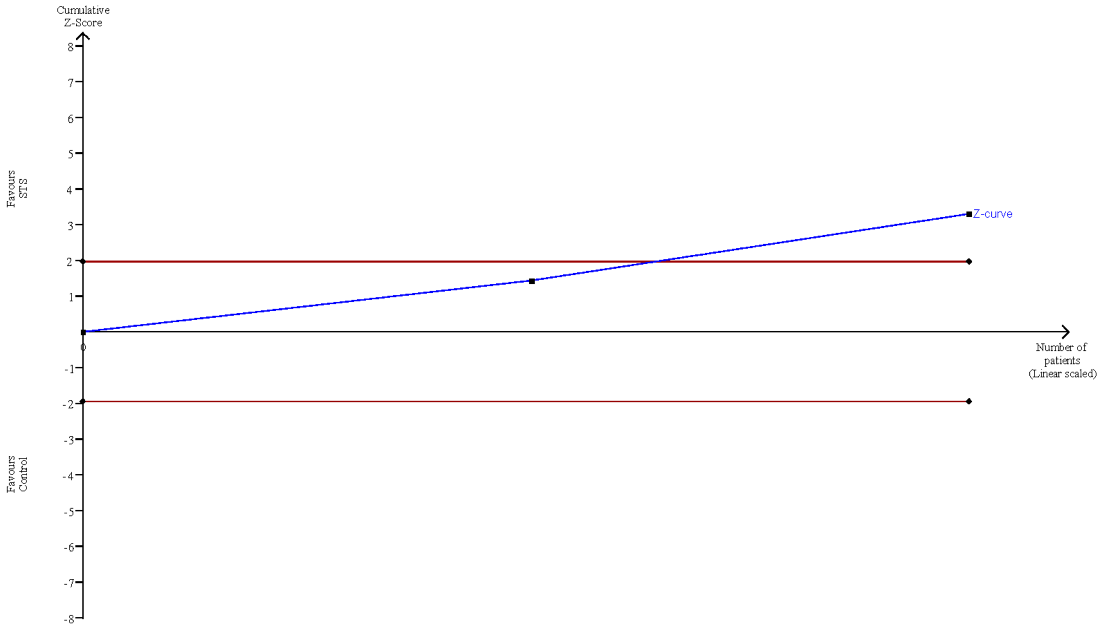

© 2021 Chen CH et al. *JAMA Network Open.*

eFigure 9. Trial Sequential Analysis (TSA) of the Association in the Older Age Group

The cumulative Z-curve did not surpass both the traditional significance boundary and the sequential monitoring boundary. Yet, the z-curve did not reach the RIS and inner wedge of futility, which mean the non-significant result remained inconclusive.

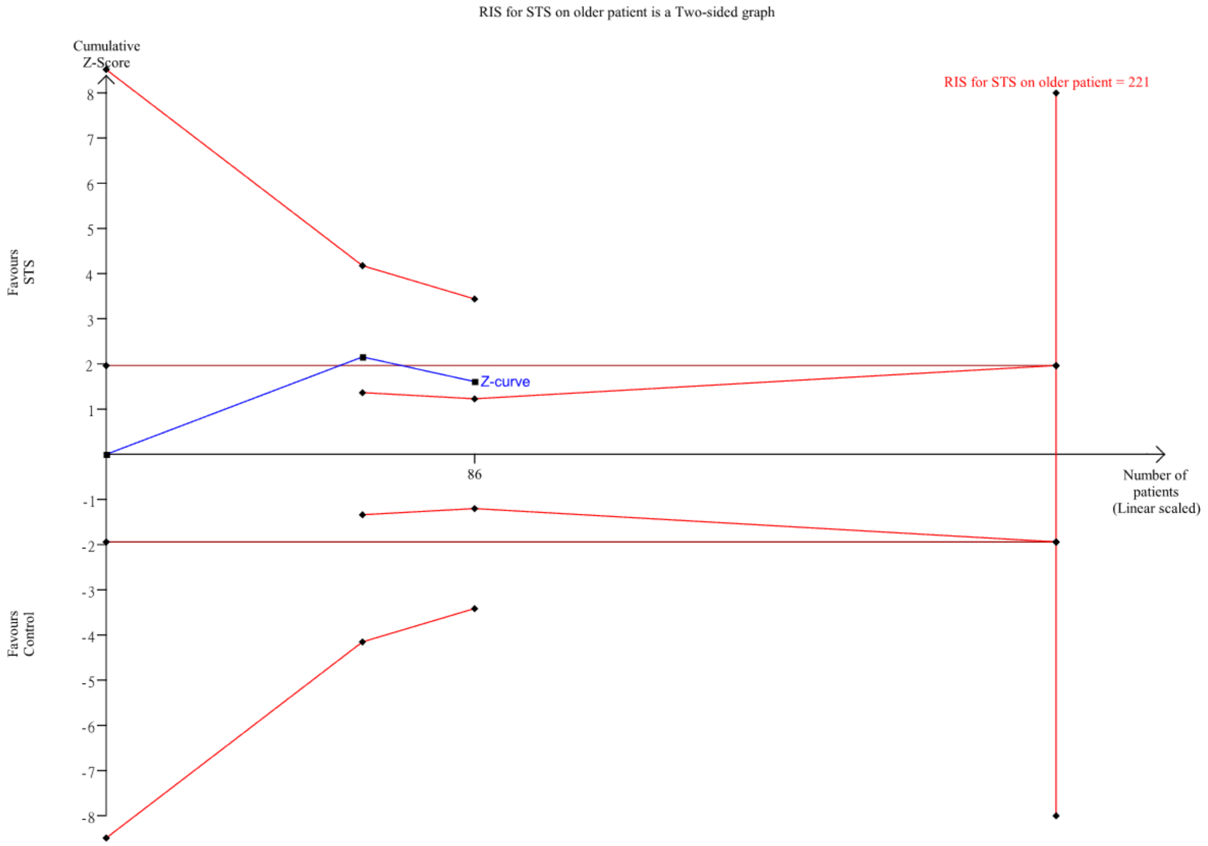

© 2021 Chen CH et al. *JAMA Network Open.*

eFigure 10. Association of Sodium Thiosulfate (STS) With Neutropenia
The pooled effect estimate showed nonsignificant difference in the development of neutropenia (RR, 1.00; 95% CI, 0.78 to 1.29; *P*=.97; I^2=0%).

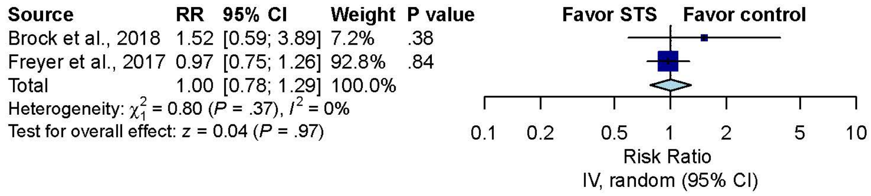

eFigure 11. Association of Sodium Thiosulfate (STS) With Thrombocytopenia
The pooled effect estimate showed a lower risk but a nonsignificant difference in the development of neutropenia (RR, 0.94; 95% CI, 0.63 to 1.42; *P*=.78; I^2=0%).

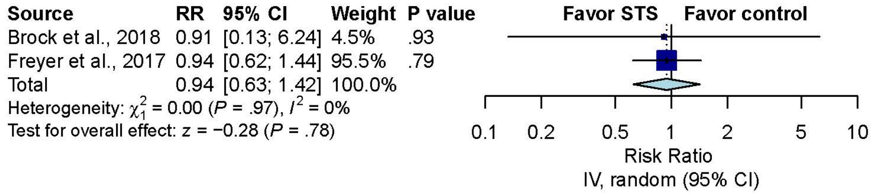

eFigure 12. Association of Sodium Thiosulfate (STS) With Anemia
The pooled effect estimate showed a lower risk but a nonsignificant difference in the development of anemia (RR, 0.88; 95% CI, 0.54 to 1.44; *P*=.61; I^2=6%).

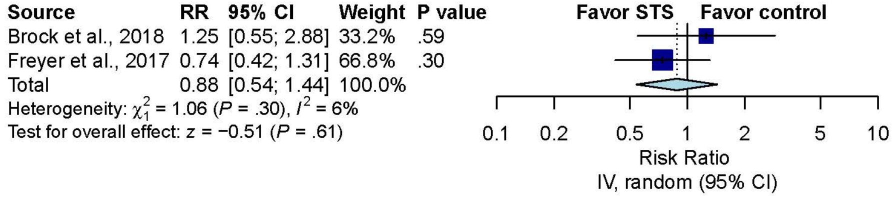

© 2021 Chen CH et al. *JAMA Network Open.*

eFigure 13. Trial Sequential Analysis (TSA) of the Association of Sodium Thiosulfate (STS) With Neutropenia

The cumulative Z-curve reached the estimated RIS and the cumulative Z-curve did not surpass either the traditional significance boundary or the sequential monitoring boundary.

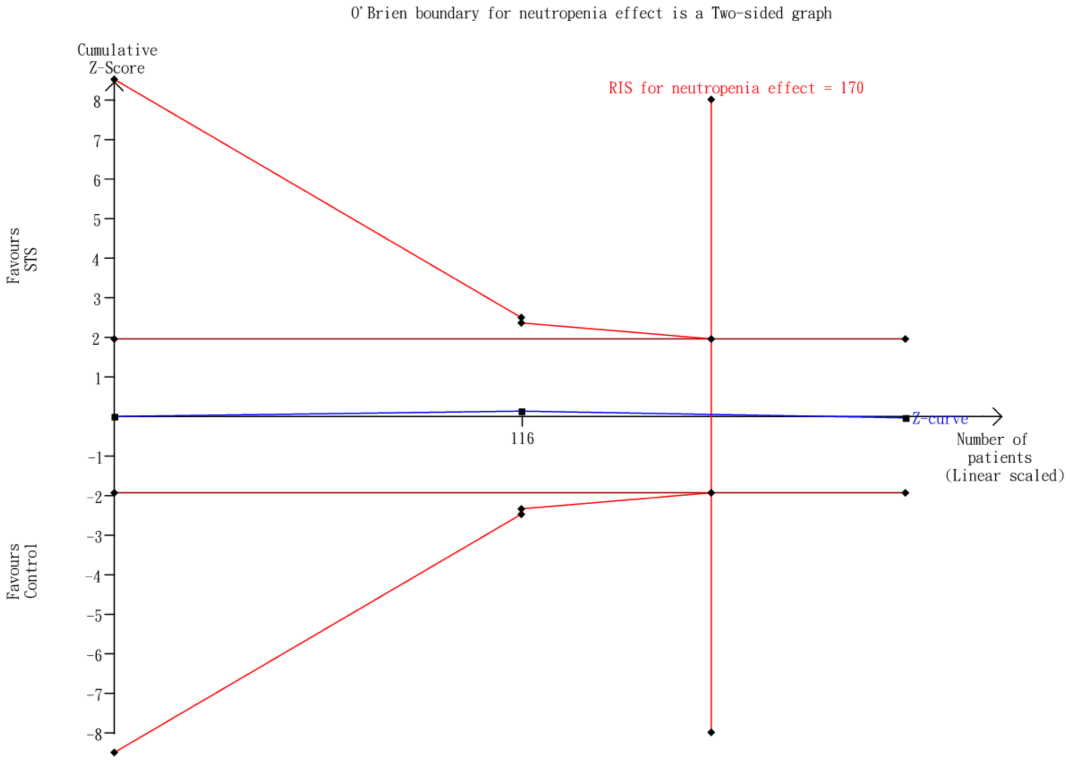

© 2021 Chen CH et al. *JAMA Network Open.*

eFigure 14. Trial Sequential Analysis (TSA) of the Association of Sodium Thiosulfate (STS) With Anemia

The cumulative Z-curve did not surpass either the traditional significance boundary or the sequential monitoring boundary and the pooled result reached the inner wedge of futility, which mean that it was conclusive for the non-significant result between these two groups.

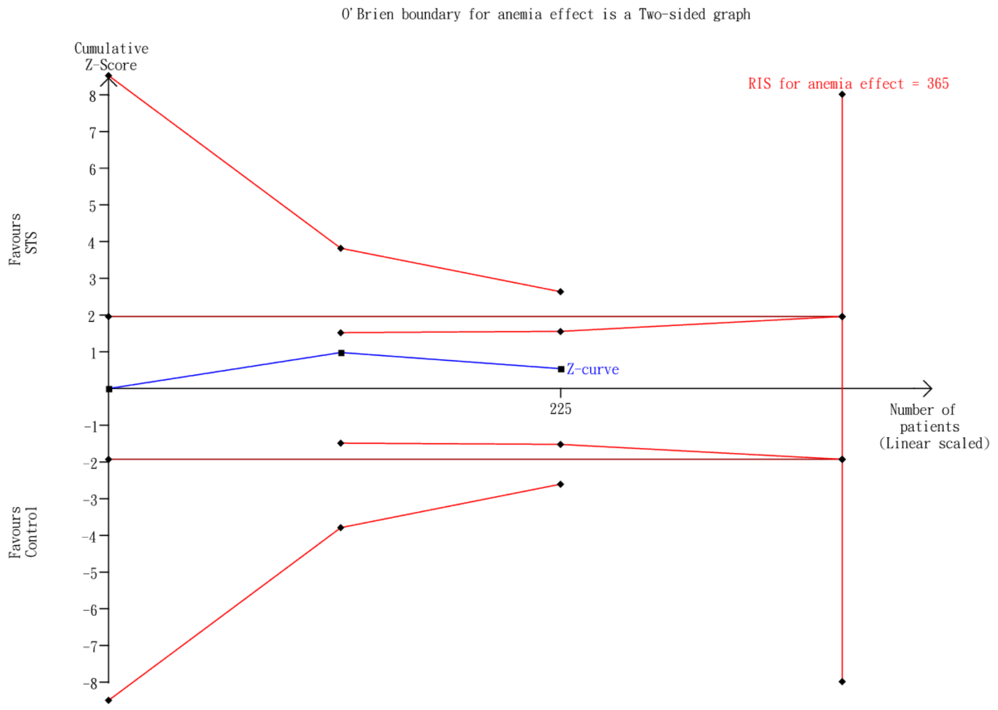

© 2021 Chen CH et al. *JAMA Network Open.*

eFigure 15. Trial Sequential Analysis (TSA) of the Association of Sodium Thiosulfate (STS) With Thrombocytopenia

The cumulative Z-curve reached the estimated RIS and the cumulative Z-curve did not surpass either the traditional significance boundary or the sequential monitoring boundary.

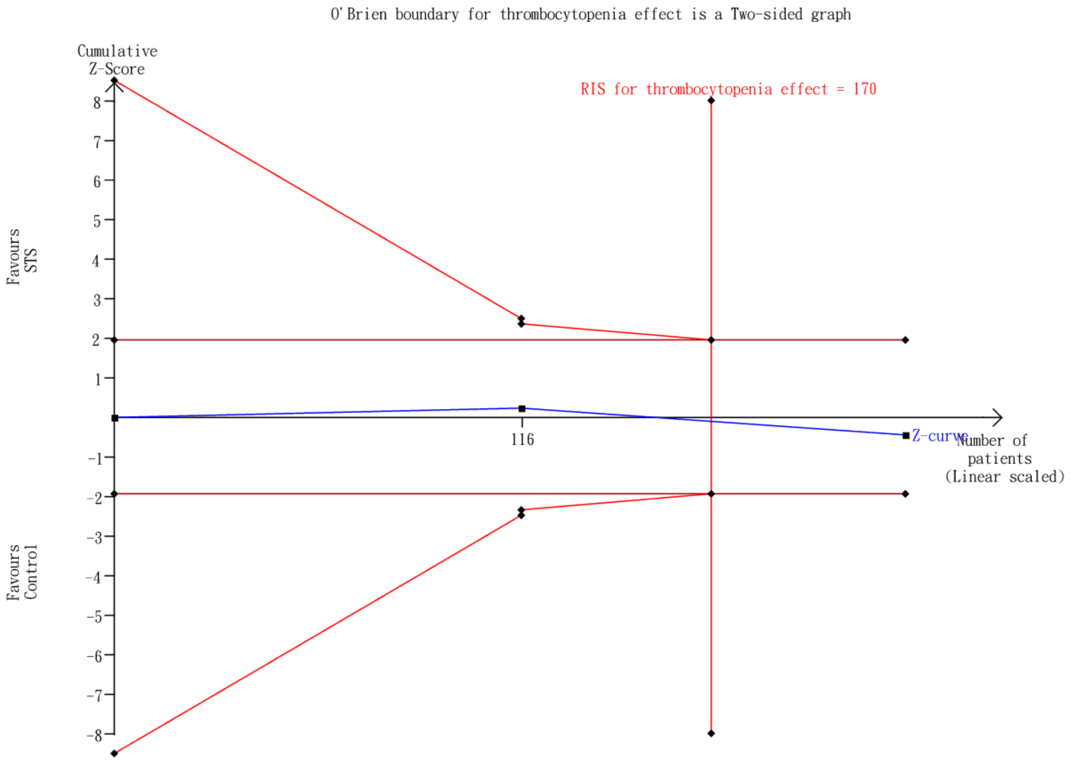

© 2021 Chen CH et al. *JAMA Network Open.*

eFigure 16. Sensitivity Analysis After Removal Of Non–Randomized Clinical Trial (RCT)

After excluding the non-RCT study, the overall effect of STS in the remaining three studies remained as a significantly lower risk for the development of ototoxicity (RR, 0.66; 95% CI, 0.50 to 0.88; P=.004; I^2=12%).

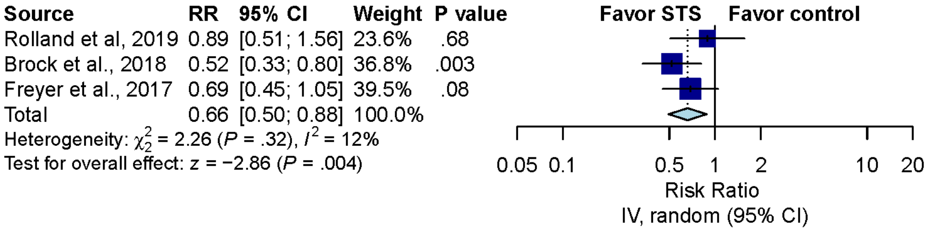

eFigure 17. Sensitivity Analysis After Removal of Study Using Carboplatin-Based Chemotherapy

After excluding the study using carboplatin-based chemotherapy, the overall effect of STS in the remaining three studies remained as a significantly lower risk for the development of ototoxicity (RR, 0.66; 95% CI, 0.50 to 0.88; P=.004; I^2=12%).

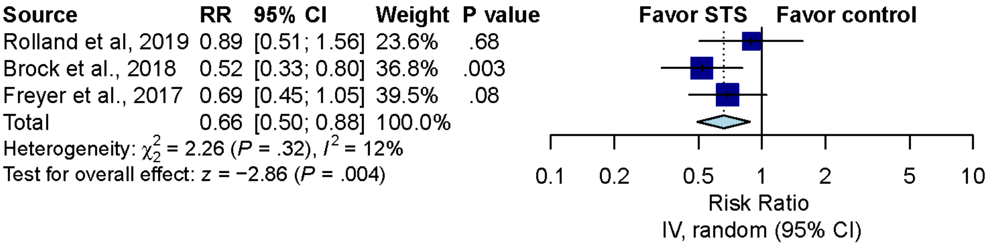

© 2021 Chen CH et al. *JAMA Network Open.*

eFigure 18. Sensitivity Analysis After Removal of Study Using Brock Grading System

After excluding the study using Brock grading system, the overall effect of STS in the remaining three studies remained as a significantly lower risk for the development of ototoxicity (RR, 0.65; 95% CI, 0.49 to 0.87; *P*=.003; I^2=18%).

Source	RR	95% CI	Weight	P value
Rolland et al, 2019	0.89	[0.51; 1.56]	22.7%	.68
Freyer et al., 2017	0.69	[0.45; 1.05]	36.9%	.08
Doolittle et al., 2001	0.52	[0.35; 0.78]	40.4%	.001
Total	0.65	[0.49; 0.87]	100.0%	

Heterogeneity: χ^2_2 = 2.43 (*P* = .30), I^2 = 18%

Test for overall effect: *z* = −2.93 (*P* = .003)

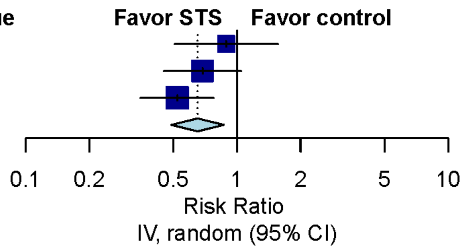

eFigure 19. Sensitivity Analysis After Removal of Study With Small Sample Size

After excluding the study with small sample, the overall effect of STS in the remaining three studies remained as a significantly lower risk for the development of ototoxicity (RR, 0.57; 95% CI, 0.45 to 0.73; *P*<.001; I^2=0%).

Source	RR	95% CI	Weight	P value
Brock et al., 2018	0.52	[0.33; 0.80]	30.3%	.003
Freyer et al., 2017	0.69	[0.45; 1.05]	32.9%	.08
Doolittle et al., 2001	0.52	[0.35; 0.78]	36.9%	.001
Total	0.57	[0.45; 0.73]	100.0%	

Heterogeneity: χ^2_2 = 1.13 (*P* = .57), I^2 = 0%

Test for overall effect: *z* = −4.56 (*P* < .001)

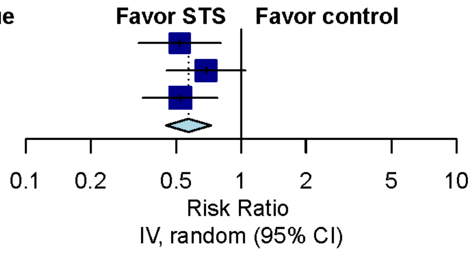

© 2021 Chen CH et al. *JAMA Network Open.*

eFigure 20. Trial Sequential Analysis (TSA) of the Sensitivity Analysis After Removal of Non–Randomized Controlled Trial (RCT)
The cumulative Z-curve surpassed the traditional significance boundary. The sequential monitoring boundary since the sample size of first study has reached estimated RIS.

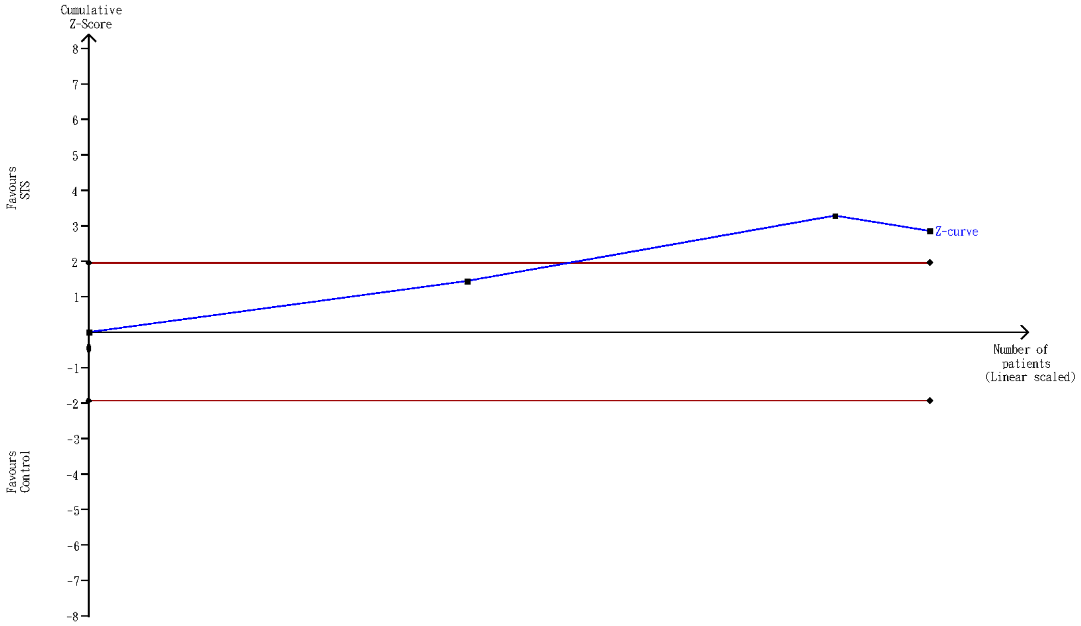

© 2021 Chen CH et al. *JAMA Network Open.*

eFigure 21. Trial Sequential Analysis (TSA) of Sensitivity Analysis After Removal Of Study Using Carboplatin-Based Chemotherapy
The cumulative Z-curve surpassed the traditional significance boundary. The sequential monitoring boundary since the sample size of first study has reached estimated RIS.

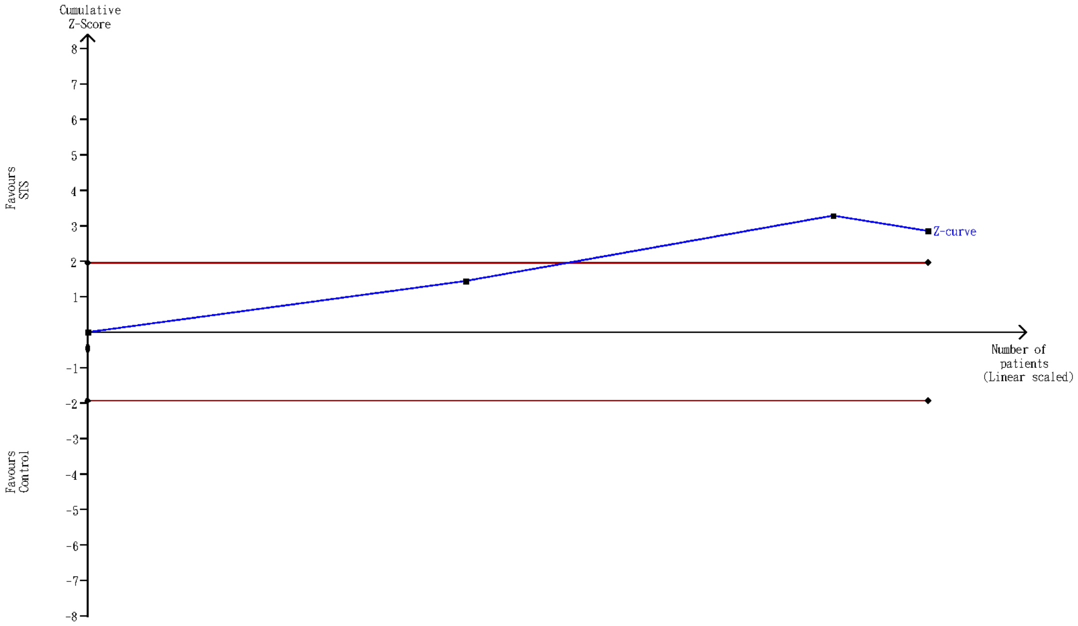

© 2021 Chen CH et al. *JAMA Network Open.*

eFigure 22. Trial Sequential Analysis (TSA) of Sensitivity Analysis After Removal of Study Using Brock Grading System

The cumulative Z-curve reached the estimated RIS and surpassed both the traditional significance boundary in and the sequential monitoring boundary of the adjusted significance threshold.

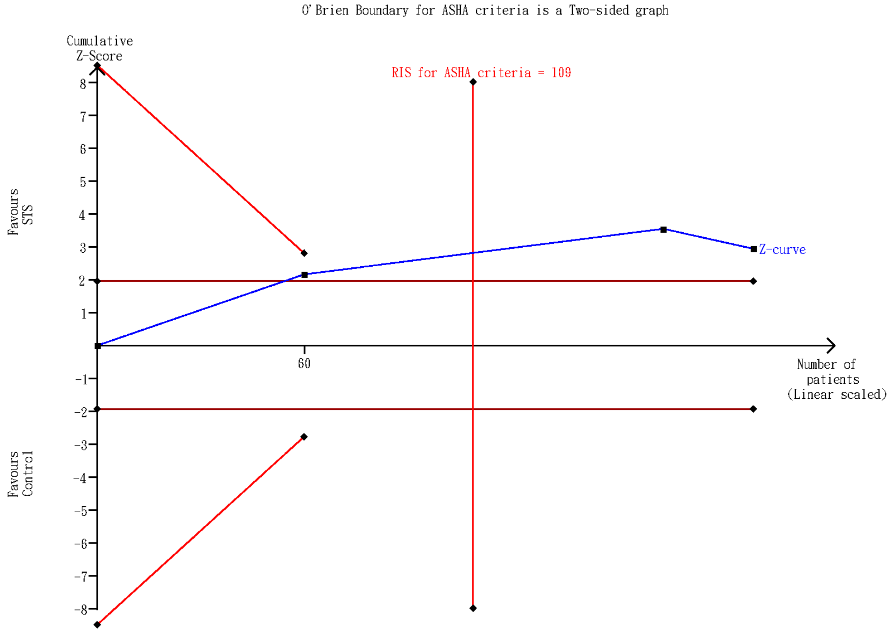

© 2021 Chen CH et al. *JAMA Network Open.*

eFigure 23. Trial Sequential Analysis (TSA) of Sensitivity Analysis After Removal of Study With Small Sample Size

The cumulative Z-curve reached the estimated RIS and surpassed both the traditional significance boundary in and the sequential monitoring boundary of the adjusted significance threshold.

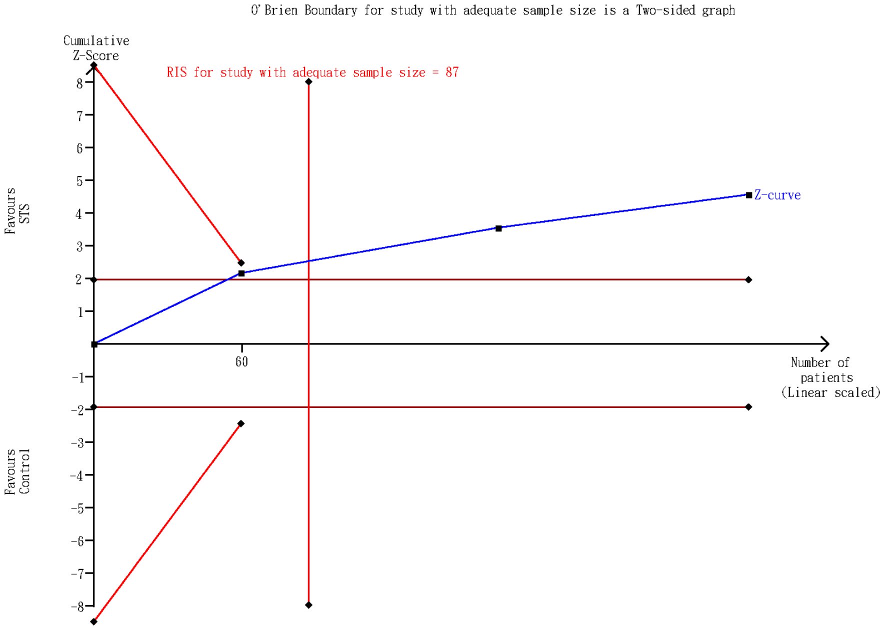

© 2021 Chen CH et al. *JAMA Network Open.*

eFigure 24. Influence Analysis of Overall Association of Sodium Thiosulfate (STS)

After leaving out each included study one at a time, the pooled estimates remained within the 95% CI of the overall pooled results for these outcomes.

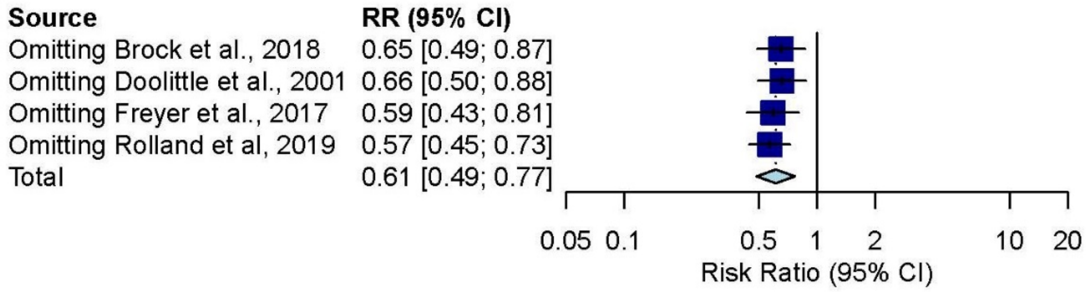

eFigure 25. Sensitivity Analysis of Sodium Thiosulfate (STS) Association With Neutropenia, Cycle 2

After pooling the effect estimates of cycle 2 of chemotherapy in the study by Freyer et al., the results remained nonsignificant for the development of neutropenia (RR, 1.06; 95% CI, 0.82 to 1.38; *P*=.67; I^2=0%).

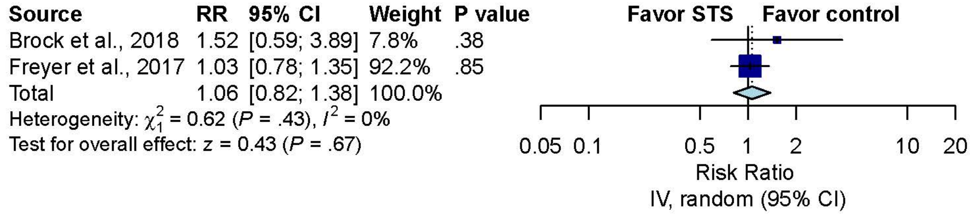

eFigure 26. Sensitivity Analysis of Sodium Thiosulfate (STS) Association With Neutropenia, Cycle 3

After pooling the effect estimates of cycle 3 of chemotherapy in the study by Freyer et al., the results remained nonsignificant for the development of neutropenia (RR, 0.98; 95% CI, 0.73 to 1.32; *P*=.88; I^2=0%).

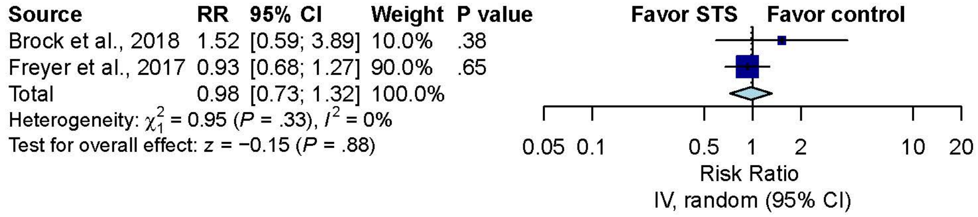

© 2021 Chen CH et al. *JAMA Network Open.*

eFigure 27. Sensitivity Analysis of Sodium Thiosulfate (STS) Association With Neutropenia, Cycle 4

After pooling the effect estimates of cycle 4 of chemotherapy in the study by Freyer et al., the results remained nonsignificant for the development of neutropenia (RR, 1.27; 95% CI, 0.87 to 1.84; *P*=.22; I^2=0%).

Source	RR	95% CI	Weight	P value
Brock et al., 2018	1.52	[0.59; 3.89]	16.0%	.38
Freyer et al., 2017	1.22	[0.81; 1.84]	84.0%	.34
Total	1.27	[0.87; 1.84]	100.0%	

Heterogeneity: χ_1^2 = 0.17 (*P* = .68), I^2 = 0%

Test for overall effect: *z* = 1.23 (*P* = .22)

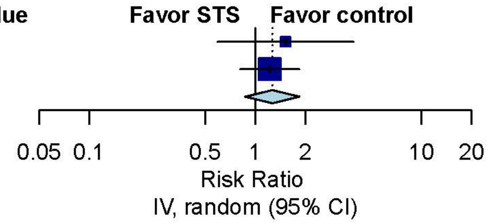

eFigure 28. Sensitivity Analysis of Sodium Thiosulfate (STS) Association With Neutropenia, Cycle 5

After pooling the effect estimates of cycle 5 of chemotherapy in the study by Freyer et al., the results remained nonsignificant for the development of neutropenia (RR, 1.18; 95% CI, 0.75 to 1.86; *P*=.48; I^2=0%).

Source	RR	95% CI	Weight	P value
Brock et al., 2018	1.52	[0.59; 3.89]	23.4%	.38
Freyer et al., 2017	1.09	[0.65; 1.83]	76.6%	.74
Total	1.18	[0.75; 1.86]	100.0%	

Heterogeneity: χ_1^2 = 0.37 (*P* = .54), I^2 = 0%

Test for overall effect: *z* = 0.71 (*P* = .48)

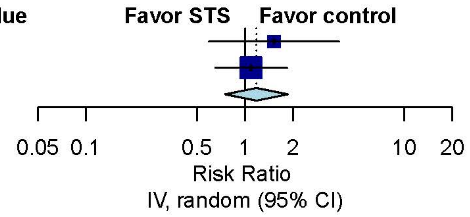

eFigure 29. Sensitivity Analysis of Sodium Thiosulfate (STS) Association With Neutropenia, Cycle 6

After pooling the effect estimates of cycle 6 of chemotherapy in the study by Freyer et al., the results remained nonsignificant for the development of neutropenia (RR, 1.28; 95% CI, 0.58 to 2.82; *P*=.54; I^2=0%).

Source	RR	95% CI	Weight	P value
Brock et al., 2018	1.52	[0.59; 3.89]	71.2%	.38
Freyer et al., 2017	0.83	[0.19; 3.65]	28.8%	.81
Total	1.28	[0.58; 2.82]	100.0%	

Heterogeneity: χ_1^2 = 0.45 (*P* = .50), I^2 = 0%

Test for overall effect: *z* = 0.61 (*P* = .54)

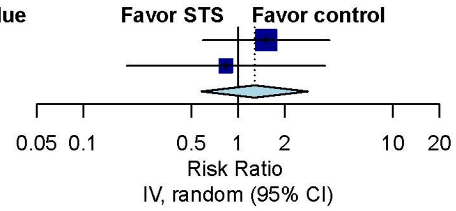

© 2021 Chen CH et al. *JAMA Network Open.*

eFigure 30. Sensitivity Analysis of Sodium Thiosulfate (STS) Association With Thrombocytopenia, Cycle 2

After pooling the effect estimates of cycle 2 of chemotherapy in the study by Freyer et al., the results remained nonsignificant for the development of thrombocytopenia (RR, 1.26; 95% CI, 0.86 to 1.85; *P*=.24; I^2=0%).

Source	RR	95% CI	Weight	P value
Brock et al., 2018	2.67	[0.39; 18.26]	4.0%	.32
Freyer et al., 2017	1.22	[0.82; 1.81]	96.0%	.32
Total	1.26	[0.86; 1.85]	100.0%	

Heterogeneity: χ^2_1 = 0.61 (*P* = .44), I^2 = 0%

Test for overall effect: z = 1.18 (*P* = .24)

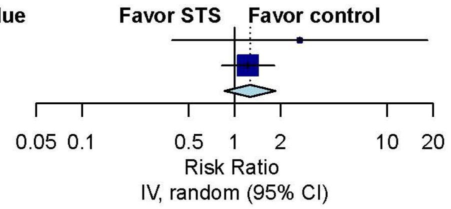

eFigure 31. Sensitivity Analysis of Sodium Thiosulfate (STS) Association With Thrombocytopenia, Cycle 3

After pooling the effect estimates of cycle 3 of chemotherapy in the study by Freyer et al., the results remained nonsignificant for the development of thrombocytopenia (RR, 1.21; 95% CI, 0.81 to 1.82; *P*=.35; I^2=0%).

Source	RR	95% CI	Weight	P value
Brock et al., 2018	0.91	[0.13; 6.24]	4.4%	.93
Freyer et al., 2017	1.23	[0.81; 1.86]	95.6%	.33
Total	1.21	[0.81; 1.82]	100.0%	

Heterogeneity: χ^2_1 = 0.09 (*P* = .77), I^2 = 0%

Test for overall effect: z = 0.94 (*P* = .35)

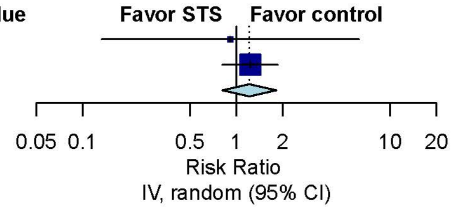

eFigure 32. Sensitivity Analysis of Sodium Thiosulfate (STS) Association With Thrombocytopenia, Cycle 4

After pooling the effect estimates of cycle 4 of chemotherapy in the study by Freyer et al., the results remained nonsignificant for the development of thrombocytopenia (RR, 1.02; 95% CI, 0.67 to 1.56; *P*=.91; I^2=0%).

Source	RR	95% CI	Weight	P value
Brock et al., 2018	0.91	[0.13; 6.24]	4.7%	.93
Freyer et al., 2017	1.03	[0.67; 1.58]	95.3%	.89
Total	1.02	[0.67; 1.56]	100.0%	

Heterogeneity: χ^2_1 = 0.01 (*P* = .90), I^2 = 0%

Test for overall effect: z = 0.11 (*P* = .91)

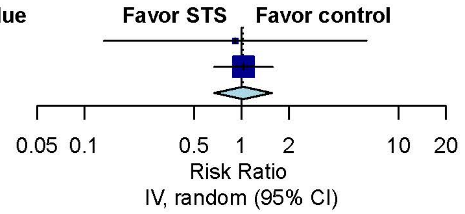

© 2021 Chen CH et al. *JAMA Network Open.*

eFigure 33. Sensitivity Analysis of Sodium Thiosulfate (STS) Association With Thrombocytopenia, Cycle 5

After pooling the effect estimates of cycle 5 of chemotherapy in the study by Freyer et al., the results remained nonsignificant for the development of thrombocytopenia (RR, 1.43; 95% CI, 0.78 to 2.61; *P*=.25; I^2=0%).

Source	RR	95% CI	Weight	P value
Brock et al., 2018	0.91	[0.13; 6.24]	9.8%	.93
Freyer et al., 2017	1.50	[0.80; 2.82]	90.2%	.21
Total	1.43	[0.78; 2.61]	100.0%	

Heterogeneity: χ^2_1 = 0.23 (*P* = .63), I^2 = 0%
Test for overall effect: *z* = 1.16 (*P* = .25)

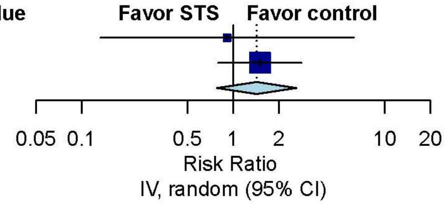

eFigure 34. Sensitivity Analysis of Sodium Thiosulfate (STS) Association With Thrombocytopenia, Cycle 6

After pooling the effect estimates of cycle 6 of chemotherapy in the study by Freyer et al., the results remained nonsignificant for the development of thrombocytopenia (RR, 0.86; 95% CI, 0.27 to 2.78; *P*=.80; I^2=0%).

Source	RR	95% CI	Weight	P value
Brock et al., 2018	0.91	[0.13; 6.24]	37.0%	.93
Freyer et al., 2017	0.83	[0.19; 3.64]	63.0%	.81
Total	0.86	[0.27; 2.78]	100.0%	

Heterogeneity: χ^2_1 = 0.01 (*P* = .94), I^2 = 0%
Test for overall effect: *z* = −0.25 (*P* = .80)

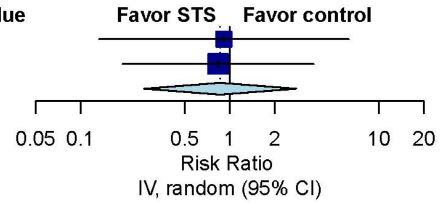

eFigure 35. Sensitivity Analysis of Sodium Thiosulfate (STS) Association With Anemia, Cycle 2

After pooling the effect estimates of cycle 2 of chemotherapy in the study by Freyer et al., the results remained nonsignificant for the development of anemia (RR, 1.03; 95% CI, 0.69 to 1.55; *P*=.88; I^2=0%).

Source	RR	95% CI	Weight	P value
Brock et al., 2018	1.25	[0.55; 2.88]	23.9%	.59
Freyer et al., 2017	0.97	[0.61; 1.55]	76.1%	.90
Total	1.03	[0.69; 1.55]	100.0%	

Heterogeneity: χ^2_1 = 0.28 (*P* = .60), I^2 = 0%
Test for overall effect: *z* = 0.16 (*P* = .88)

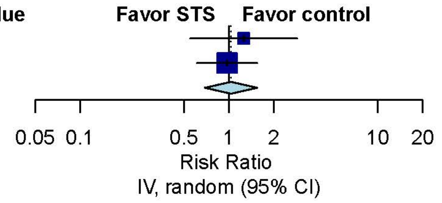

© 2021 Chen CH et al. *JAMA Network Open.*

eFigure 36. Sensitivity Analysis of Sodium Thiosulfate (STS) Association With Anemia, Cycle 3

After pooling the effect estimates of cycle 3 of chemotherapy in the study by Freyer et al., the results remained nonsignificant for the development of anemia (RR, 1.21; 95% CI, 0.77 to 1.90; *P*=.40; I^2=0%).

Source	RR	95% CI	Weight	P value
Brock et al., 2018	1.25	[0.55; 2.88]	29.4%	.59
Freyer et al., 2017	1.19	[0.70; 2.04]	70.6%	.52
Total	1.21	[0.77; 1.90]	100.0%	

Heterogeneity: χ^2_1 = 0.01 (*P* = .92), I^2 = 0%
Test for overall effect: *z* = 0.84 (*P* = .40)

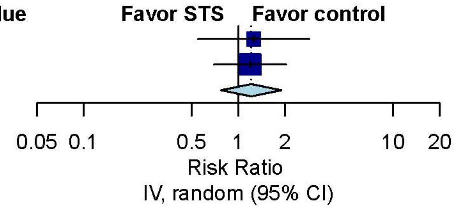

eFigure 37. Sensitivity Analysis of Sodium Thiosulfate (STS) Association With Anemia, Cycle 4

After pooling the effect estimates of cycle 4 of chemotherapy in the study by Freyer et al., the results remained nonsignificant for the development of anemia (RR, 0.96; 95% CI, 0.61 to 1.51; *P*=.87; I^2=0%).

Source	RR	95% CI	Weight	P value
Brock et al., 2018	1.25	[0.55; 2.88]	29.6%	.59
Freyer et al., 2017	0.86	[0.50; 1.48]	70.4%	.59
Total	0.96	[0.61; 1.51]	100.0%	

Heterogeneity: χ^2_1 = 0.55 (*P* = .46), I^2 = 0%
Test for overall effect: *z* = −0.16 (*P* = .87)

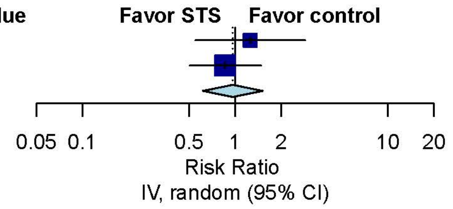

eFigure 38. Sensitivity Analysis of Sodium Thiosulfate (STS) Association With Anemia, Cycle 5

After pooling the effect estimates of cycle 5 of chemotherapy in the study by Freyer et al., the results remained nonsignificant for the development of anemia (RR, 1.36; 95% CI, 0.91 to 2.04; *P*=.13; I^2=0%).

Source	RR	95% CI	Weight	P value
Brock et al., 2018	1.25	[0.55; 2.88]	23.6%	.59
Freyer et al., 2017	1.40	[0.88; 2.22]	76.4%	.15
Total	1.36	[0.91; 2.04]	100.0%	

Heterogeneity: χ^2_1 = 0.05 (*P* = .82), I^2 = 0%
Test for overall effect: *z* = 1.51 (*P* = .13)

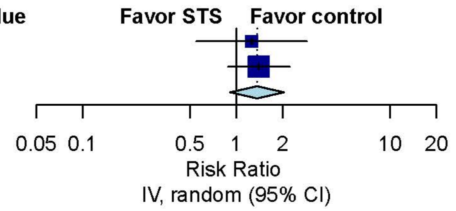

© 2021 Chen CH et al. *JAMA Network Open.*

eFigure 39. Sensitivity Analysis of Sodium Thiosulfate (STS) Association With Anemia, Cycle 6

After pooling the effect estimates of cycle 6 of chemotherapy in the study by Freyer et al., the results remained nonsignificant for the development of anemia (RR, 1.09; 95% CI, 0.53 to 2.24; *P*=.81; I^2=0%).

Source	RR	95% CI	Weight	P value
Brock et al., 2018	1.25	[0.55; 2.88]	75.2%	.59
Freyer et al., 2017	0.71	[0.17; 3.03]	24.8%	.65
Total	1.09	[0.53; 2.24]	100.0%	

Heterogeneity: χ^2_1 = 0.44 (*P* = .51), I^2 = 0%

Test for overall effect: z = 0.24 (*P* = .81)

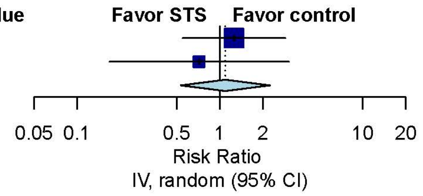

© 2021 Chen CH et al. *JAMA Network Open.*

eReferences.

1. Brock PR, Maibach R, Childs M, et al. Sodium Thiosulfate for Protection from Cisplatin-Induced Hearing Loss. Clinical Trial, Phase III; Journal Article; Multicenter Study; Randomized Controlled Trial; Research Support, N.I.H., Extramural; Research Support, Non-U.S. Gov't. *New England journal of medicine*. 2018;378(25):2376-2385. doi:10.1056/NEJMoa1801109
2. Doolittle ND, Muldoon LL, Brummett RE, et al. Delayed sodium thiosulfate as an otoprotectant against carboplatin-induced hearing loss in patients with malignant brain tumors. *Clin Cancer Res*. Mar 2001;7(3):493-500.
3. Freyer DR, Chen L, Krailo MD, et al. Effects of sodium thiosulfate versus observation on development of cisplatin-induced hearing loss in children with cancer (ACCL0431): a multicentre, randomised, controlled, open-label, phase 3 trial. Clinical Trial, Phase III; Comparative Study; Journal Article; Multicenter Study; Observational Study; Randomized Controlled Trial; Research Support, N.I.H., Extramural. *The lancet Oncology*. 2017;18(1):63-74. doi:10.1016/S1470-2045(16)30625-8
4. Rolland V, Meyer F, Guitton MJ, et al. A randomized controlled trial to test the efficacy of trans-tympanic injections of a sodium thiosulfate gel to prevent cisplatin-induced ototoxicity in patients with head and neck cancer. *J Otolaryngol Head Neck Surg*. Jan 16 2019;48(1):4. doi:10.1186/s40463-019-0327-x

© 2021 Chen CH et al. *JAMA Network Open.*
